# A Closed‐Loop Hybrid Discovery System of Type I Photosensitizers for Hypoxic Tumor Therapy

**DOI:** 10.1002/advs.202515103

**Published:** 2025-12-12

**Authors:** Xia Ling, Yixin Zhu, Min Li, Zongliang Xie, Lei Cao, Wentao Song, Dandan Wang, Duo Mao, Xiaonan Wang, Bin Liu

**Affiliations:** ^1^ Joint School of National University of Singapore and Tianjin University International Campus of Tianjin University Binhai New City Fuzhou 350207 China; ^2^ Department of Chemical and Biomolecular Engineering National University of Singapore Singapore 117585 Singapore; ^3^ Institute of Precision Medicine The First Affiliated Hospital of Sun Yat‐Sen University Sun Yat‐Sen University Guangzhou 510080 China; ^4^ Department of Chemical Engineering Tsinghua University Beijing 100084 China

**Keywords:** closed‐loop hybrid discovery system, hypoxic tumor therapy, machine learning, Type І photosensitizer

## Abstract

Type I photosensitizers (PSs), which operate effectively under low‐oxygen conditions, offer a promising approach to overcome hypoxia‐associated challenges in solid tumor therapy. However, their design remains challenging due to the limited number of reported molecules with diverse structures, as well as insufficient understanding of the underlying mechanisms. Herein, a closed‐loop hybrid discovery system is developed that combines molecular excited‐state calculations with machine learning (ML) to rationally design and predict high‐performance Type I PSs for hypoxic tumor therapy. Through a support vector machine (SVM) classification model, 664 potential Type I PSs are identified from a molecular space based on donor‐acceptor (D‐A) and donor‐acceptor‐donor (D‐A‐D) structures. Among these, two candidates, M1 and M2, are synthesized and experimentally verified as Type I PSs, exhibiting aggregate‐induced enhancement of Type I reactive oxygen species (ROS) generation. Both in vitro and in vivo studies demonstrated their ability to induce intracellular Type I ROS generation and effectively suppress tumor growth. The work highlights the potential of ML in the design and prediction of Type I PSs for hypoxic tumor therapy.

## Graphical abstract

The work developed a closed‐loop hybrid discovery system to rationally design and predict high‐performance Type I PSs for hypoxic tumor therapy. 664 Potential candidates are identified from a dataset through a support vector machine (SVM) classification model, and two candidates are experimentally verified as Type I PSs, which highlighted the potential ofmachine learning (ML) in the design of Type I PSs.

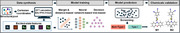

## 1 Introduction

Photodynamic therapy (PDT) is a promising and precise cancer treatment modality known for its high spatiotemporal selectivity and minimal invasiveness.^[^ ^1^ ^]^ PDT involves photochemical reactions between PSs and tissue‐endogenous oxygen under appropriate light excitation, generating cytotoxic ROS that effectively kill cancer cells.^[^ ^2^ ^]^ Traditional PSs are predominantly Type II agents that produce singlet oxygen (^1^O_2_) via energy transfer with heavy reliance on oxygen, which limits their therapeutic efficacy in the hypoxic microenvironment of solid tumors (oxygen pressure < 5 mm Hg).^[^ ^3^ ^]^ In comparison, Type I PSs represent a compelling alternative, producing superoxide anion radical (O_2_^−•^) and hydroxyl radical (HO•) through electron transfer with partial oxygen circulation.^[^ ^4^ ^]^ This low oxygen dependence enables Type I PSs to mitigate the effects of tumor hypoxia, improving their PDT performance.^[^ ^5^ ^]^ Despite their advantages, the rational design of Type I PSs remains a significant challenge due to the intense competition between electron transfer and energy transfer pathways.^[^ ^6^ ^]^ To address this, several strategies have been employed to regulate the photochemical reactions of PSs: i) Reducing the energy gaps between the lowest singlet excited state (S_1_) and adjacent triplet excited states (T_n_) ($\Delta E_{\left(S_{1}-T_{n}\right)}$), or increasing their spin‐orbit coupling (SOC) constants through heavy‐atom effect, facilitates the intersystem crossing (ISC) process, ultimately promoting the formation of triplet excited‐state PSs (^3^PSs^*^).^[^ ^7^, ^8^ ^]^ ii) Designing PSs with low ground‐state reduction potentials (below −0.33 V vs. NHE for O_2_ + e^−^ → O_2_^−•^) ensures their thermodynamical feasibility to reduce O_2_ into O_2_^−•^.^[^ ^9^, ^10^, ^11^ ^]^ iii) Reducing the energy gaps between the lowest triplet excited state (T_1_) and ground state (S_0_) ($\Delta E_{\left(T_{1}-S_{0}\right)}$) of PSs below the energy thresholds for ^1^O_2_ formation (first excited state ^1^Δ_g_ ↔ ^3^Σ_g_^−^: 0.98 eV, second excited state ^1^Σ_g_^+^ ↔ ^3^Σ_g_^−^: 1.63 eV), which prevents energy transfer to oxygen, thus suppressing Type II ROS generation.^[^ ^12^, ^13^, ^14^, ^15^ ^]^ Despite these advancements, challenges persist.^[^ ^16^ ^]^ Enhancing ISC may inadvertently facilitate the Type II pathway, which competes with Type I.^[^ ^17^, ^18^ ^]^ Additionally, fine‐tuning the reduction potential is complicated due to the unclear structure‐property relationships. Reducing $\Delta E_{\left(T_{1}-S_{0}\right)}$ of PSs often necessitates the design of large‐conjugated molecular structures that are difficult to synthesize.^[^ ^19^ ^]^ Given these limitations, a more comprehensive approach based on the modulation of excited‐state properties is needed. Excited‐state properties encompass not only low energy levels (e.g., S_1_, T_1_), which govern ISC and energy transfer, but also higher energy levels that significantly impact photochemical reaction pathways. However, the limited number and irregular structure types of reported Type I PSs hinder the development of tools for Type I PS design by regulating excited‐state properties. Moreover, traditional trial‐and‐error methods are insufficient to meet the growing demand for efficient Type I PSs, emphasizing the urgent need for innovative approaches to accelerate their discovery.

Machine learning (ML)‐assisted molecular design and discovery, combined with computational chemistry, offer a promising alternative to traditional labor‐intensive trial‐and‐error methods for developing high‐performance PSs without relying on predefined empirical rules.^[^ ^20^, ^21^ ^]^ In this regard, Buglak et al. developed a quantitative structure‐property relationship (QSPR) model aimed at accurately predicting ^1^O_2_ quantum yields in porphyrins and heavy‐atom‐free BODIPY PSs.^[^ ^22^, ^23^ ^]^ Additionally, our group previously proposed a self‐improving discovery system that integrates first‐principle‐based computations with active learning and Bayesian optimization.^[^ ^24^ ^]^ This system accelerates the identification of high‐performance PSs and continuously refines the predictive model as more data are generated. Despite these advancements, current ML‐assisted platforms focus on screening Type II PSs and lack attention to Type I PSs. The structural diversity of potential Type I PSs poses additional challenges, complicating the development of reliable ML models for their accurate discovery and instant prediction. To overcome this, selecting a suitable research object with tunable structures and modifiable excited‐state properties is crucial. To date, reported Type I PSs mainly include antennae‐fullerene conjugates,^[^ ^25^ ^]^ triarylmethanes,^[^ ^26^ ^]^ benzophenothiazine analogues,^[^ ^27^ ^]^ donor‐acceptor (D‐A)^[^ ^28^ ^]^ and donor‐acceptor‐donor (D‐A‐D) conjugates.^[^ ^29^ ^]^ Among these, D‐A and D‐A‐D conjugates stand out as ideal candidates due to their synthetic feasibility, small singlet‐triplet state energy gaps ($\Delta E_{\left(S_{1}-T_{n}\right)}$), and ease of structural and excited‐state properties modulations.^[^ ^30^ ^]^ With the intense D‐A strength and extended π‐bridges, they usually exhibit strong intramolecular charge transfer (ICT) characteristics, leading to the separated HOMO‐LUMO distribution and promoting the ISC process.^[^ ^31^, ^32^ ^]^ Leveraging these properties, an ML model based on D‐A and D‐A‐D conjugates is a prospective tool for the rational design and accurate prediction of Type I PSs, accelerating the development of next‐generation PSs for PDT.

**Scheme 1:**
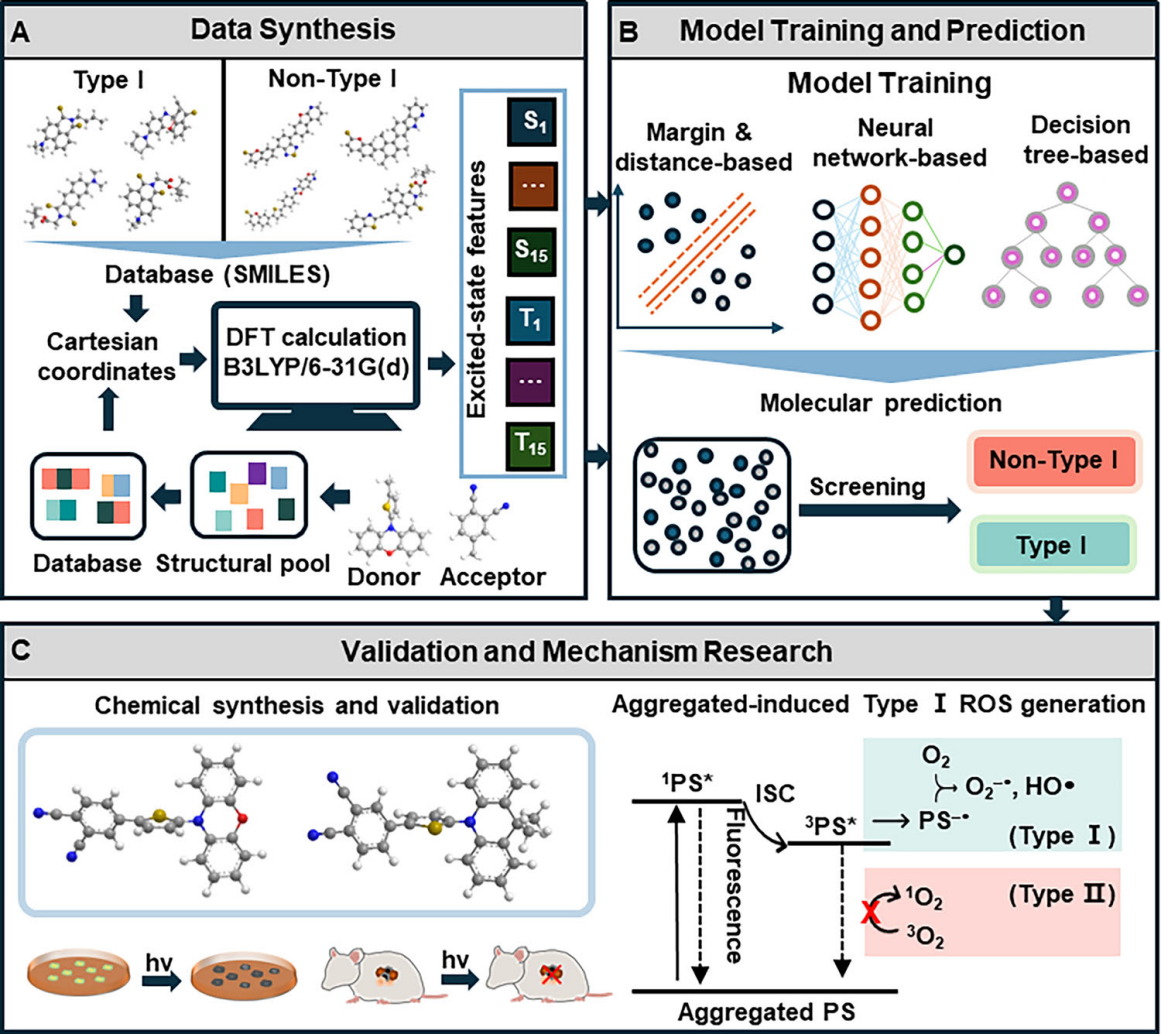
Illustration of the ML‐driven discovery process of Type I PSs, encompassing data synthesis, model training and molecular prediction, molecular synthesis and verification, and mechanism exploration. A) Data synthesis process for reported Type I and non‐Type I PSs, and candidates generated by molecular reformulation. Molecules in SMILES format undergo TD‐DFT calculations to extract key excited‐state properties, including energy levels of singlet (S_1_‐S_15_) and triplet (T_1_‐T_15_) excited states. B) The training of classification models based on excited‐state features using margin‐based, neural network‐based, and decision tree‐based algorithms, to distinguish Type I and non‐Type I PSs. C) From the predicted candidate set of potential Type I PSs, two molecules were selected, synthesized, and validated as Type I PSs with aggregated‐induced Type I ROS enhancement for in vitro and in vivo cancer therapy.

Herein, motivated by the absence of techniques for specifically identifying Type I PSs, we developed a closed‐loop hybrid discovery system based on D‐A and D‐A‐D structures to efficiently learn and predict high‐performance Type I PSs for hypoxic tumor therapy. As illustrated in **Scheme** **1**, this approach leveraged the excited‐state properties of Type I and non‐Type I PSs, obtained via time‐dependent density functional theory (TD‐DFT) calculations, to train a model capable of accurately and quickly predicting the Type I properties of PSs using five ML algorithms. By reformulating electron‐rich donors and electron‐deficient acceptors, we constructed a comprehensive molecular dataset with varied excited‐state properties to identify 664 potential Type I PSs via a support vector machine (SVM) classification model with high prediction accuracy. From this candidate set, two molecules, M1 and M2, were selected based on their structural novelty, synthetic accessibility, and small $\Delta E_{\left(S_{1}-T_{n}\right)}$, and large $\Delta E_{\left(T_{1}-S_{0}\right)}$. M1 and M2 were successfully synthesized and experimentally verified as Type I PSs, exhibiting aggregate‐enhanced Type I ROS generation, attributed to their aggregate‐induced ISC enhancement. TD‐DFT calculations of M2 single crystal revealed that synergistic intramolecular and intermolecular charge transfer interactions in its aggregated state resulted in denser energy‐level distributions and smaller $\Delta E_{\left(S_{1}-T_{n}\right)}$ compared to its monomeric form, thereby facilitating ISC transition. Subsequently, due to its superior efficiency in generating O_2_^−•^ compared to the commercial photosensitizer Rose Bengal (RB), M2 was further evaluated for in vitro and in vivo PDT on hypoxic tumors, demonstrating effective intracellular Type I ROS generation and tumor growth suppression. These results highlighted the potential of ML‐driven approaches for the efficient design and precise prediction of Type I PSs, paving the way for advanced PDT applications in challenging hypoxic tumor environments.

## 2 Results and Discussion

### 2.1 Selection of Embedding Vectors for Representation

Due to the mechanism of ROS generation, the excited‐state properties of PSs significantly influence the efficiency and pathway of subsequent photochemical reactions. Through ML, we can analyze excited‐state properties of molecules obtained through TD‐DFT calculations, thereby more accurately and reliably identifying potential Type I PSs and further exploring more design strategies. A suitable computational representation of the excited‐state properties of PSs is crucial for advancing ML methods in Type I PS design and screening. Traditional molecular representation methods (such as Morgan circular fingerprint, atom‐pair fingerprint, and topological torsion fingerprint) often rely on conventional molecular fingerprints, which do not adequately capture the dynamic nature of molecules in excited states. Therefore, our study proposes an approach that utilizes excited‐state properties (energy levels of S_1_‐S_15_ and T_1_‐T_15_) as descriptors for studying photosensitized reactions and predicting Type I PSs. This approach aims to enhance understanding and optimization of photosensitized processes by accurately representing the dynamic nature of molecules in excited states. Furthermore, most of the reported Type І PSs are conjugated structures and exhibit different solubilities in organic solvents and aqueous solution, existing in molecular and aggregated states, respectively. They often perform different ROS generation behaviors at molecular and aggregated states, which is another important impact for Type І PSs study and prediction. Aromatic ring count (AR), having an explicit effect on the compound's aqueous solubility, is an easily collected parameter for model training.^[^ ^33^ ^]^ Therefore, a structural feature associated with molecular packing tendencies (AR) is also incorporated to complement the excited‐state descriptors, to account for potential aggregation effects.

### 2.2 Molecular Data Collection and Excited‐State Property Analysis

A comprehensive molecular library of 10081 PSs based on D‐A and D‐A‐D structures was first compiled and bifurcated into two distinct sets: a training dataset comprising Type I and non‐Type I PSs served as the foundational learning platform for the ML models, and a potential dataset was designed to identify the potential Type I PSs. Based on the different mechanistic pathways of electron transfer and energy transfer in photosensitization reactions, the training dataset was manually curated, which identified PSs as either Type I or non‐Type I based on their dominant ROS generation mechanism (detailed in the section on Collection of Training D−ataset at the Experimental Section). All molecular structures were standardized, and duplicated structures were removed via RDKit. Additionally, to facilitate a more reliable model, we make efforts to ensure a balanced representation of these two classes to avoid biased learning outcomes. The molecules in the potential dataset were constructed using a series of donors and acceptors to ensure molecular diversity (molecular generation method is detailed in the Supporting Information experimental section). Ground state optimization and excited‐state energy calculations (S_1_‐S_15_, T_1_‐T_15_) were performed by using TD‐DFT with B3LYP functional and 6‐31G(d) basis set for all molecules in the molecular library.^[^ ^24^, ^34^ ^]^ The consistent computational approach is crucial to maintain uniformity and comparability within the dataset, especially for those molecules whose excited‐state properties were unreported in the previous literature and were designed by us. This systematic and balanced approach enhances the logical consistency of the ML model.

### 2.3 Machine Learning Classifiers and Feature Significance

**Figure 1:**
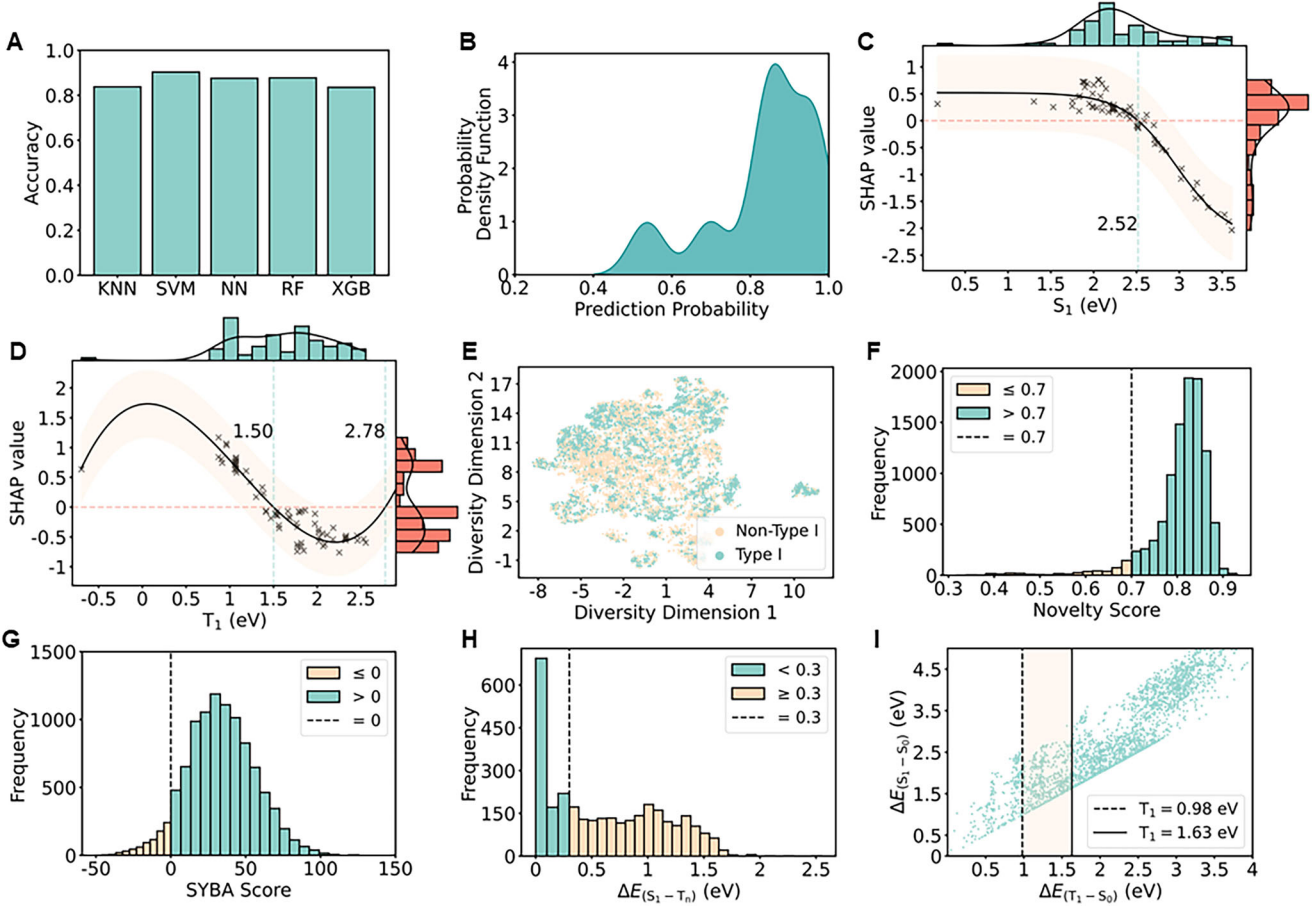
A) A bar chart comparing the accuracy of five ML algorithms: K‐Nearest Neighbors (KNN), Support Vector Machine (SVM), Neural Networks (NN), Random Forest (RF), and XGBoost. Each bar represents cross‐validation accuracy. B) Density distribution of predicted probabilities for the Type I test dataset. C, D) Partial SHAP dependence plots for two representative features, S_1_ (C) and T_1_ (D). The data points are displayed as a scatter plot, with the mean trend indicated by the black line and the standard deviation (SD) shaded around it. Dashed lines mark the cutoff value for the variable. The cutoff values were selected based on inflection points in SHAP value trends and correlated with physical transitions in photophysical properties associated with Type I PS performance. Adjacent to each plot, histograms depict the distribution of the SHAP values and the corresponding variable values, providing a visual summary of the spread and density of data. E) The UMAP plot shows the spatial distribution of Type I (light green) and non‐Type I (dull yellow) molecules. F) Distribution of novelty score for generated molecules. The novelty score = 1 ‐ Tanimoto Similarity. A molecule with a higher novelty score indicates that it has lower structural similarity with reported Type I PSs. G) Distribution of SYBA score. Molecules with positive scores are considered easy to synthesize. H) The histogram of energy gaps between S_1_ and T_n_ ($\Delta E_{\left(S_{1}-T_{n}\right)}$) for identified Type I PSs. “T_n_” represents the triplet state that has the closest energy level to the energy level of the S_1_ state. I) The scatter plots of energy gaps between T_1_ and S_0_ ($\Delta E_{\left(T_{1}-S_{0}\right)}$) of potential Type I PS candidates.

An array of ML classification models was employed to predict the categorization of PSs as Type I. Five distinct ML algorithms, including k‐nearest neighbors (KNN), support vector machine (SVM), neural network (NN), random forest (RF), and extreme gradient boosting (XGBoost), were selected to encompass a broad spectrum of predictive paradigms, enhancing the reliability of the findings.^[^ ^35^, ^36^, ^37^, ^38^ ^]^ The key to these models is the unique representation of PSs through an embedding vector, formed by concatenating calculated properties from S_1_ to S_15_ and T_1_ to T_15_ energy states. This embedding vector served as input for the ML models, ensuring consistent and comprehensive consideration of excited‐state properties. Following the embedding vector formulation, the models were optimized to capture the influence of hyperparameters on performance, ensuring reliable predictive accuracy (The hyperparameters that yielded the best performance are summarized in Table S1, Supporting Information). The empirical findings revealed that the SVM classification model is the most proficient, outperforming the other models with an overall cross‐validation accuracy of 90.3% (**Figure** **1**A). On the independent test dataset, the model achieved an accuracy of 89.5%, providing an evaluation of its performance on unseen data. We further investigated the prediction confidence on the Type I predictions. The predicted probabilities that can be seen are toward 1 (Figure 1B), confirming the reliability of the model in identifying the Type I PSs. To gain insight into the decision‐making process of the model, we performed the Shapley Additive exPlanations (SHAP) analysis.^[^ ^39^ ^]^ Among all descriptors, S_1_, T_1,_ and T_2_, emerged as the most important descriptors that contribute meaningfully to drive the model prediction (Figure S1, Supporting Information), highlighting the central role of excited‐state features in determining Type I PS activity. To further explore their influence, we focused our analysis on S_1_ and T_1_ (Figure 1C,D), which ranked highly in SHAP importance and also represent fundamental excited‐state transitions. These two plots illustrate how SHAP values vary non‐linearly with changes in S_1_ and T_1_, showing the impact of these features on the prediction result of the model. The SHAP value decreases with increasing S_1_ and shows a U‐shaped trend with T_1_, highlighting different regions of influence for each variable. We studied the specific characteristics and nuances of T_1_ within Type I PSs, aiming to elucidate the mechanistic and structural attributes that confer this critical classification attribute. In addition, we have developed a user interface for classifying Type I PSs, which allows users without a computing background to easily input molecular data and receive accurate predictions (Figure S2, Supporting Information). The interface streamlines the identification process, providing accessibility to a wider range of users while maintaining high predictive accuracy. This tool enhances the efficiency of both research and practical applications, making it an asset in the study and development of PSs.

### 2.4 Molecule Screening, Synthesis, and Characterization

**Figure 2:**
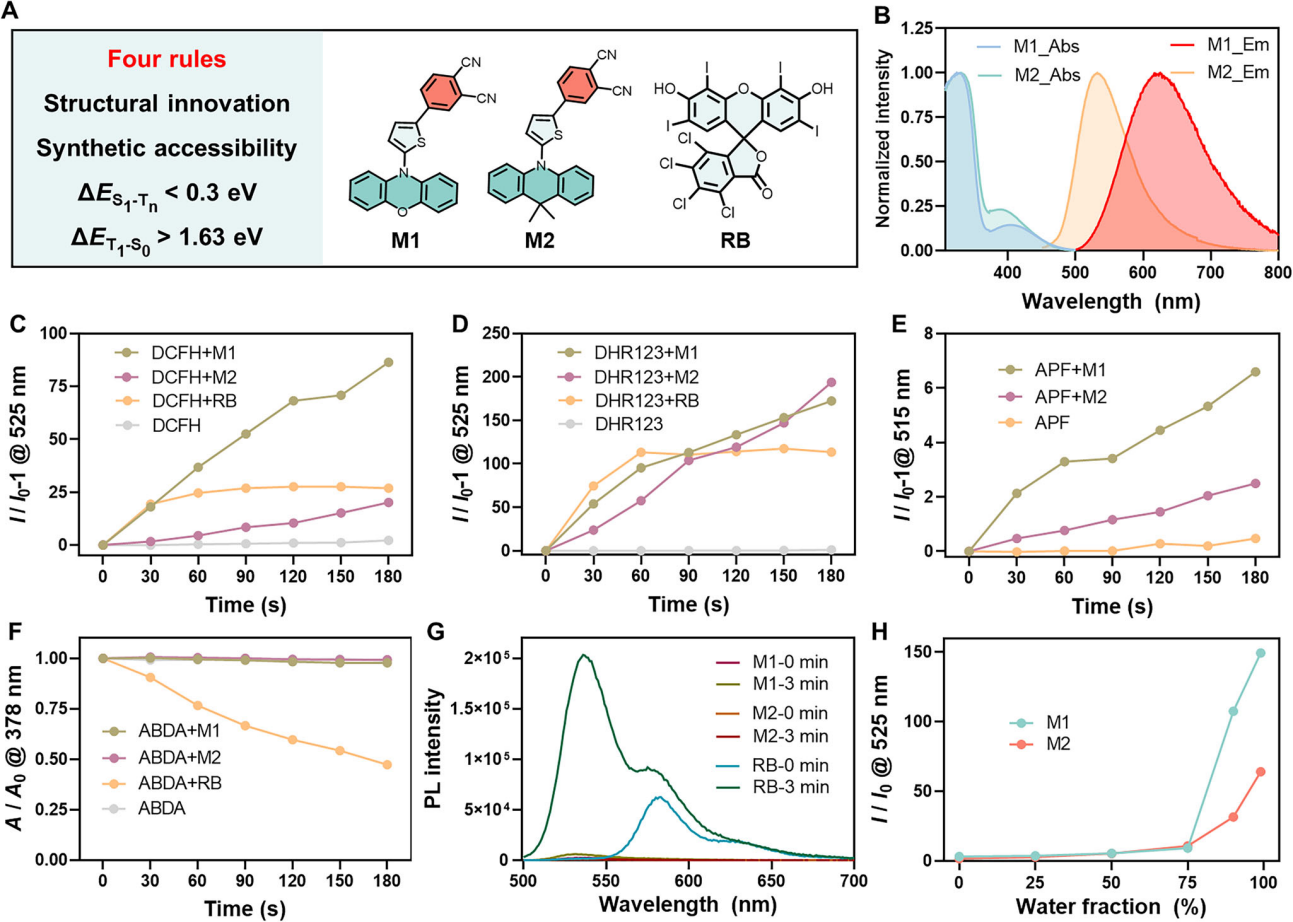
Photophysical properties and PDT performances of M1 and M2. A) Four criteria for screening Type І PS candidates from the structural pool, along with the chemical structures of M1, M2, and RB. B) Normalized absorption spectra of M1 and M2 in DMSO and normalized emission spectra of M1 and M2 in the DMSO/H_2_O (v:v = 1:99) mixture, λ_ex_ = 400 nm. C–E) Relative fluorescence changes of DCFH (C), DHR123 (D), and APF (E) with M1, M2, and RB in aqueous solution under white light irradiation (30 mW cm^−2^) for 0–3 min. F) Relative absorption changes of ABDA with M1, M2, and RB in aqueous solution under white light irradiation (30 mW cm^−2^) for 0–3 min. G) Fluorescence spectra of DHR123 with M1, M2, and RB in THF under white light irradiation for 0 and 3 min. H) Relative fluorescent intensity changes of DHR123 in the presence of M1 and M2 under white light irradiation for 3 min, measured at varying water fractions in water/THF mixtures.

Once the model is trained, the next step is to screen the chemical space to identify the most promising candidate molecules based on the predicted properties. First, the entire chemical space was examined using the Uniform Manifold Approximation and Projection (UMAP) technique to visualize the prediction dataset (Figure 1E, with detailed algorithmic parameters summarized in Table S2, Supporting Information).^[^ ^40^ ^]^ Using molecular fingerprints, we plotted two classes: Type I PSs and non‐Type I PSs. Despite significant overlap between these classes, our model successfully distinguished Type I PSs from chemically similar molecules by using excited‐state properties as feature inputs. Next, these potential candidates were evaluated based on their four characteristics: structural innovation, synthetic accessibility, and small $\Delta E_{\left(S_{1}-T_{n}\right)}$, and large $\Delta E_{\left(T_{1}-S_{0}\right)}$: i) To explore more new Type I PSs which can help to understand the underlying mechanism, candidates that have rarely or not been reported from previous studies were screened out first. The novelty score is assessed based on Tanimoto similarity. The histogram in Figure 1F shows the screened novelty scores, indicating the level of uniqueness in the molecular dataset relative to reported Type I PSs.^[^ ^41^ ^]^ Specifically, the novelty score was calculated as 1 minus the maximum Tanimoto similarity between each candidate molecule and the known Type I PSs in the training set. Although the donor and acceptor fragments are derived from known substructures, novel combination patterns can result in significantly different topological structures, leading to high novelty scores even when using familiar building blocks. The distribution shown in Figure 1F thus reflects both fragment novelty and scaffold‐level diversity. ii) The synthetic accessibility and cost‐effectiveness of candidate molecules were evaluated to prioritize those with straightforward synthesis routes. To facilitate this, we applied the SYBA (Bayesian estimation of synthetic accessibility) scoring method, as shown in Figure 1G, revealing that 92.03% of candidates were classified as easily synthesizable.^[^ ^42^ ^]^ iii) Candidates with a small energy gap of $\Delta E_{\left(S_{1}-T_{n}\right)}$ (< 0.3 eV) were selected (Figure 1H), which can facilitate the intersystem crossing (ISC) process, thereby promoting ROS generation.^[^ ^43^ ^]^ iv) Although lowering $\Delta E_{\left(T_{1}-S_{0}\right)}$ is beneficial to inhibit the energy transfer pathway, synthesizing PSs with a low T_1_ state is challenging due to their large π‐conjugate skeletons. Moreover, in contrast to empirical designs, most of the potential Type I PSs predicted from the molecule space have a larger $\Delta E_{\left(T_{1}-S_{0}\right)}$ than 1.63 eV, as shown in the Figure 1I. In consequence, we specifically selected candidates with a large $\Delta E_{\left(T_{1}-S_{0}\right)}$ (> 1.63 eV) to explore whether the T_1_ energy level is a critical factor influencing the energy transfer between ^3^PS^*^ and ^3^O_2_. Following this in‐depth expert knowledge analysis, we streamlined the decision‐making process by dividing it into smaller, more manageable batches, guided by predictions from our final models. Finally, this structured approach enabled the successful identification of 664 promising Type I PSs with novel structures, high synthetic feasibility, and small $\Delta E_{\left(S_{1}-T_{n}\right)}$, and large $\Delta E_{\left(T_{1}-S_{0}\right)}$. To validate the effectiveness and accuracy of the closed‐loop hybrid discovery platform, two molecules (M1 and M2) were selected further based on their high Type I probabilities derived from our best‐performed model, synthesized, and thoroughly characterized through comprehensive experiments, including organic synthesis, ROS species characterization, TD‐DFT calculations, and the assessment of PDT efficacy both in vitro and in vivo. As shown in **Figure** **2**A, the chemical structures of M1 and M2 feature phenoxazine and 9,9‐dimethyl‐9,10‐dihydroacridine donors, thiophene π‐bridges, and phthalonitrile acceptors. Detailed synthetic routes and conditions of M1, M2, and all intermediates are provided in the Experimental Section, and Supplementary Information (Scheme S1, Supporting Information), and their structures were satisfactorily characterized by NMR spectroscopy and mass spectrometry (Figures S3–S9, Supporting Information).

Subsequently, the optical properties of M1 and M2 were examined by measuring their UV–vis absorption and fluorescence emission spectra (Figure 2B; Figure S10, Supporting Information). M1 and M2 exhibit visible light absorption in DMSO and fluorescence emission in aqueous solution, with maximum emission wavelengths of 625 nm and 532 nm, respectively. The emission intensity variations of M1 and M2 in THF/water mixtures with different water fractions demonstrated their aggregate‐induced emission (AIE) behaviors (Figures S11 and S12, Supporting Information).^[^ ^44^ ^]^ To assess the predicted accuracy of the closed‐loop hybrid discovery system, the PDT performance of M1 and M2 molecules was studied in comparison to the commercial photosensitizer RB by employing different ROS indicators. First, in dichloromethane (DCM) solvent, dihydrorhodamine 123 (DHR123) was used to detect O_2_^−•^ generation. As shown in Figure S13 (Supporting Information), under white light irradiation, the fluorescence intensity of DHR123 at 525 nm in the presence of M1 and M2 increased more than control group, indicating that they can effectively induce O_2_^−•^ production. In contrast, compared to RB, M1 and M2 did not generate obvious ^1^O_2_ in DCM when 1,3‐diphenylisobenzofuran (DPBF) was utilized as the indicator (Figures S14 and S15, Supporting Information). These results indicated that M1 and M2 are both Type I PSs. Considering the practical application of PSs in a physiological environment, the PDT performances of M1 and M2 were further evaluated in aqueous solution at their aggregate states (Figure S16, Supporting Information). Under white light irradiation, the fluorescent intensity of dichlorofluorescin (DCFH) containing M1 and M2 at 525 nm significantly increased, while the control group (sole DCFH) showed minimal change, demonstrating M1 and M2 are effective PSs in aqueous solution (Figure 2C; Figure S17, Supporting Information). The 173‐fold and 195‐fold changes of relative fluorescent intensity for DHR123+M1 and DHR123+M2 in aqueous solution, respectively, indicated that they were efficient Type I PSs and exhibited higher O_2_^−•^ production than RB (Figure 2D; Figure S18, Supporting Information). Additionally, another Type I ROS species, hydroxyl radical (HO•), was characterized using aminophenyl fluorescein (APF) indicator. The fluorescence of APF with M1 and M2 displaced ≈6.5‐fold and 2.5‐fold enhanced, respectively, compared to the control group, reflecting their definite HO• generation capacities (Figure 2E; Figure S19, Supporting Information). Next, Type II ROS production of M1 and M2 in aqueous solution was studied using 9,10‐anthracenediyl‐bis(methylene)dimalonic acid (ABDA) decomposition and singlet oxygen sensor green (SOSG) fluorescence imaging methods. The absorption of the ABDA+RB group at 378 nm gradually decreased with increasing irradiation time, while the absorption of the ABDA+M1/M2 groups remained unchanged (Figure 2F; Figure S20, Supporting Information), indicating that M1 and M2 could not produce any ^1^O_2_. The result was also confirmed by the unchanged fluorescent intensity of SOSG with M1 and M2 under light irradiation, as shown in Figures S21 and S22 (Supporting Information). The comparison of Type І ROS generation between M1, M2, and Type І PS crystal violet (CV) shows that M1 and M2 are promising Type І PSs with stronger O_2_^−•^ and HO• generation capacities than CV (Figures S23–S25, Supporting Information). These results demonstrated the correct prediction of the ML model. To compare the Type І performances of M1/M2 at molecular and aggregated states, we used water/THF mixed solvent with different water fractions to regulate their aggregation. As the water fraction increased, the fluorescence of DHR123 containing M1 and M2 upon irradiation gradually increased, different from the RB positive control group (Figure 2G,H; Figures S26–S28, Supporting Information), reflecting that the aggregation of M1 and M2 enhanced their O_2_^−•^ generation efficiency. In conclusion, M1 and M2 are pure Type I PSs, producing intense O_2_^−•^ and HO• with aggregate‐enhanced Type I ROS generation, demonstrating their high potential for hypoxic tumor therapy.

### 2.5 Mechanism Study

**Figure 3:**
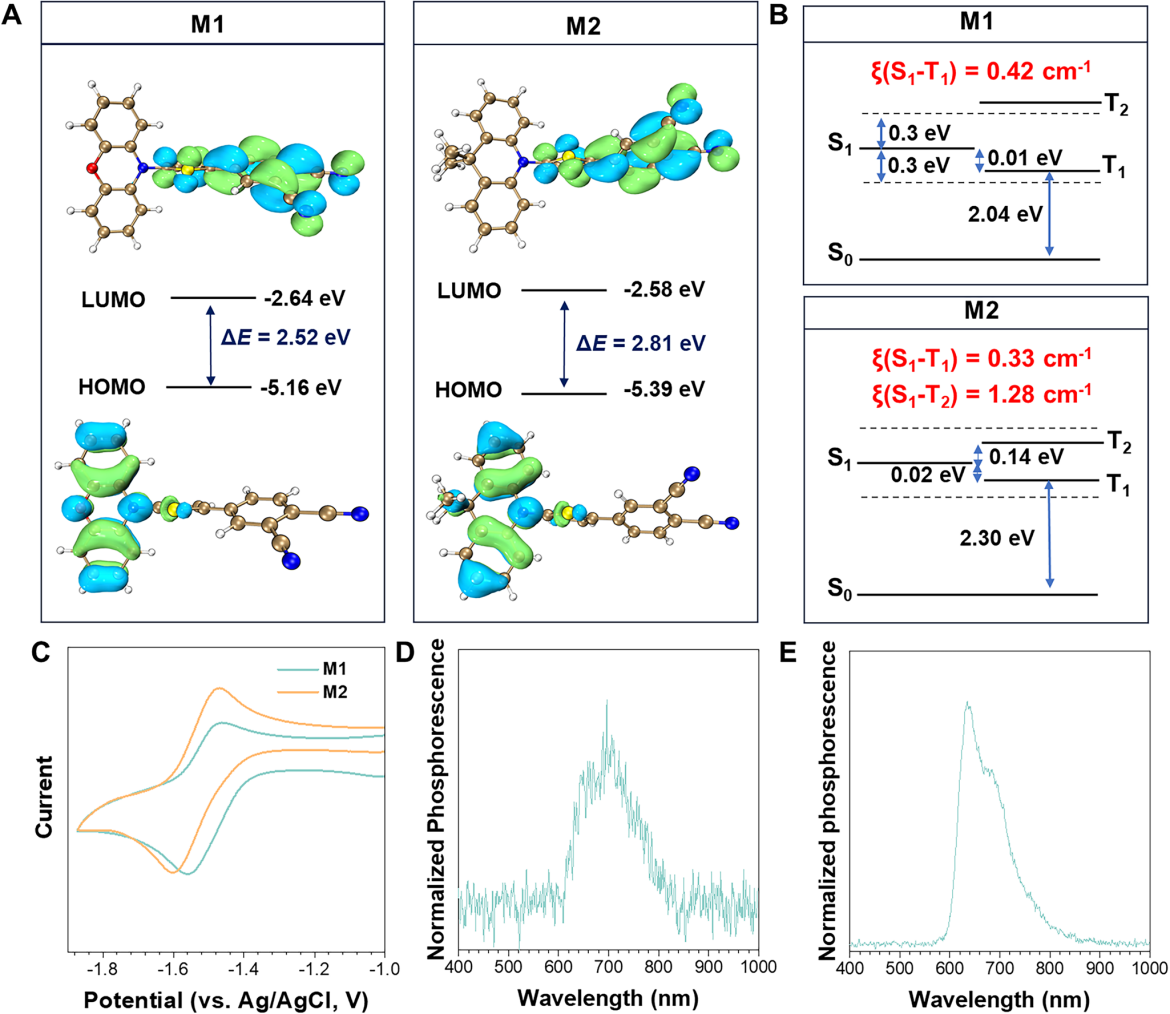
A) Molecular orbital density distributions and corresponding energy levels of M1 and M2. B) Energy levels and SOC constants of singlet and triplet excited states for M1 and M2. C) Cyclic voltammograms of M1 and M2 in dichloromethane (DCM) with 0.1 M tetrabutylammonium hexafluorophosphate ((*n*‐Bu)_4_N^+^PF_6_^−^) as the electrolyte, glassy carbon as the working electrode, Ag/AgCl as the reference electrode, Pt wire as the counter electrode, with Fc/Fc^+^ as the external reference. The reduction potentials (vs. Ag/AgCl) of M1 and M2 are −1.38 and −1.42 V, respectively, obtained from the cyclic voltammograms. The reduction potentials (vs. NHE) of M1 and M2 are −1.18 and −1.22 V, respectively. D, E) Normalized phosphorescence spectra of M1 (D) and M2 (E) at 77 K in aqueous solution.

To gain insights into the determinants underlying the Type I ROS‐generating behaviors of M1 and M2, systematic TD‐DFT calculations and analyses were carried out using Gaussian 09, ORCA, Multiwfn 3.8, and VMD 1.9.3.^[^ ^45^, ^46^ ^]^ The frontier molecular orbitals, energy levels, and spin‐orbit coupling (SOC) constants of the singlet and triplet excited states of M1 and M2 were calculated based on the optimized ground‐state geometries at the B3LYP/6‐31G(d) level. As shown in **Figure** **3**A, the electron density distributions of the lowest unoccupied molecular orbitals (LUMOs) of M1 and M2 are primarily localized on the electron‐withdrawing acceptors, while those of the highest occupied molecular orbitals (HOMOs) are mainly concentrated on the electron‐rich donors. The separated LUMO‐HOMO distributions and small LUMO‐HOMO energy gaps of M1 and M2 (2.52 and 2.81 eV, respectively) illustrate their strong intramolecular charge transfer (ICT) properties. Owing to the D‐A strength, M1 and M2 exhibit relatively small $\Delta E_{\left(S_{1}-T_{n}\right)}$ ($\Delta E_{\left(S_{1}-T_{1}\right)}$: 0.01 eV for M1, 0.02 eV for M2, $\Delta E_{\left(S_{1}-T_{2}\right)}$: 0.14 eV for M2) and large SOC (ξ(S_1_‐T_1_) = 0.42 cm^−1^ for M1; ξ(S_1_‐T_1_) = 0.33 cm^−1^ and ξ(S_1_‐T_2_) = 1.28 cm^−1^ for M2), which are expected to facilitate the formation of ^3^PS^*^ via ISC process, thereby enhancing efficient ROS generation (Figure 3B; Table S3, Supporting Information). To further evaluate the feasibility of electron transfer in Type I ROS generation, the Gibbs free energy for electron transfer between M1/M2 and oxygen was calculated using the Rehm‐Weller equation (Δ*G* = *E*_ox_ – *E*_red_ – *E*_0‐0_).^[^ ^47^ ^]^ In addition, the Gibbs free energy changes of M1 and M2 extracting an electron from the environmental hydroxyl anion were also calculated at the B3LYP/6‐31G(d) level. As shown in Table S4 (Supporting Information), these negative Gibbs free energy values indicate that M1 and M2 are thermodynamically favorable for acquiring an electron from environmental substrates and converting into intermediate radical anions (PS^−•^), which subsequently transfer an electron to O_2_, generating O_2_^−•^. Next, the ground‐state reduction potential, *E*_re_(PS/PS^−•^) of M1 and M2 was measured by cyclic voltammetry (Figure 3C). M1 and M2 have lower ground‐state reduction potential *E*_re_(PS/PS^−•^) of −1.18 and −1.22 V (vs. NHE), respectively, compared to −0.33 V (*E*_re_(O_2_/O_2_^−•^), vs. NHE), demonstrating that they are thermodynamically feasible for reducing O_2_ to O_2_^−•^. Next, we compared the $\Delta E_{\left(T_{1}-S_{0}\right)}$ of M1 and M2 with the energy gaps of ^1^O_2_ and ^3^O_2_ to assess their feasibility of ^1^O_2_ production. Based on the normalized phosphorescence spectra of M1 and M2 at 77 K (Figure 3D,E), their $\Delta E_{\left(T_{1}-S_{0}\right)}$ values were calculated to be 2.10 and 2.07 eV, respectively, both significantly higher than 1.63 eV. Despite this, neither M1 nor M2 generated detectable ^1^O_2_ in organic and aqueous solution (Figure 2F; Figrues S14, S20, and S21, Supporting Information). This suggests that energy transfer between M1/M2 and O_2_ is inhibited in both molecular and aggregated states. This is because the energy difference between the triplet excited state of PSs and oxygen is too large, rendering energy transfer between them ineffective and preventing it from competing with electron transfer.^[^ ^14^ ^]^

**Figure 4:**
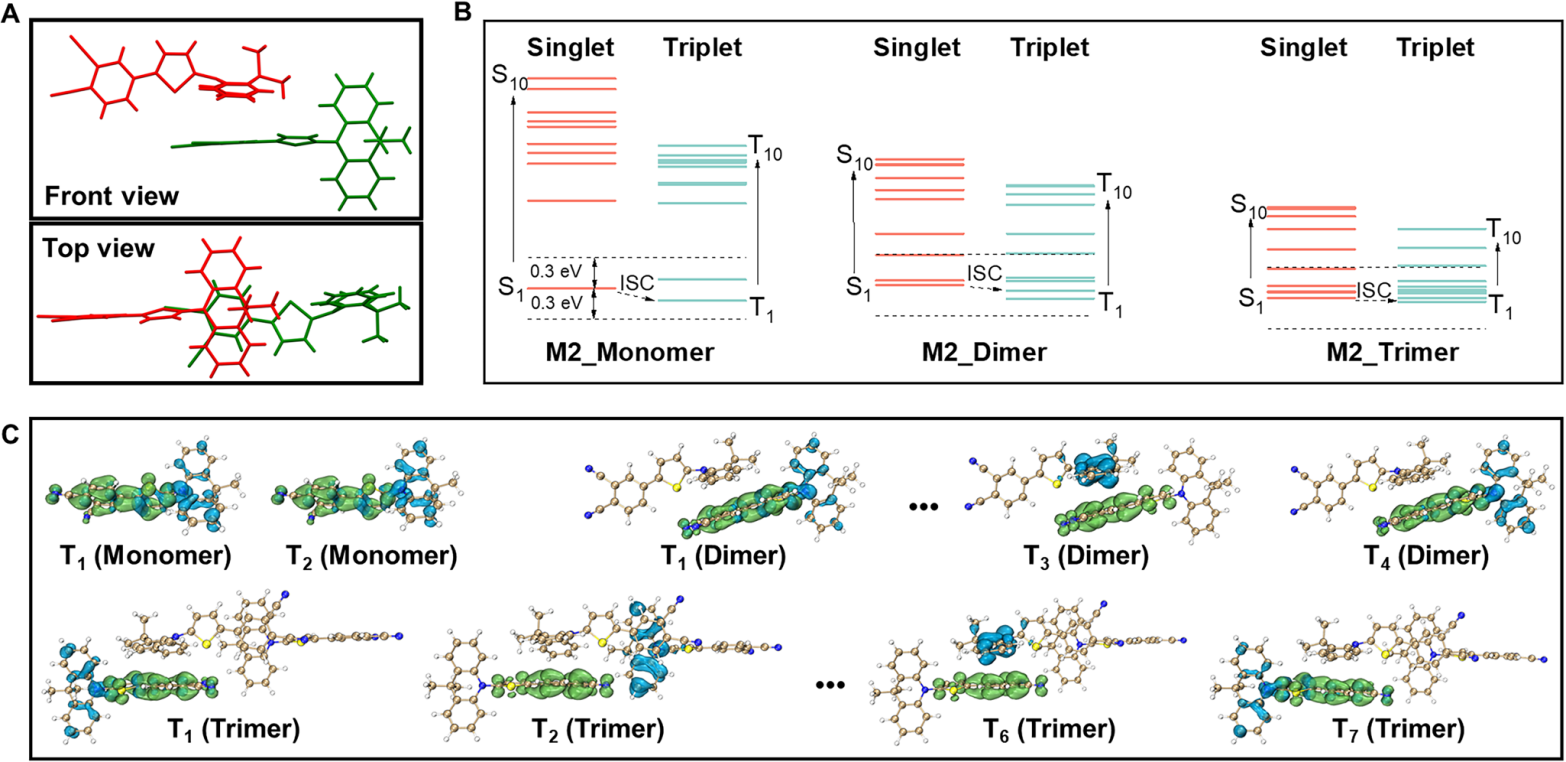
A) Stacking structure of M2_dimer extracted from its single crystal. B) Energy levels of singlet and triplet excited states of M2_monomer, M2_dimer, and M2_trimer. C) Isosurface maps of electron‐hole distributions of M2_monomer, M2_dimer, and M2_trimer in the triplet excited state. Green and blue regions represent electrons and holes, respectively.

To further elucidate the underlying mechanism of aggregate‐induced Type I ROS enhancement, TD‐DFT calculations and electron‐hole isosurface analyses on both single molecules and aggregate structures of the M2 singlet crystal in singlet and triplet states were carried out (Table S5, Supporting Information). The dimer and trimer extracted from the M2 singlet crystal are simulated as their aggregated state, while the monomer is simulated as its molecular state. The packing structures of M2 are shown in **Figures** **4**A and S29 (Supporting Information), where the donor of one molecule forms noteworthy *π–π* stacking with the acceptor of an adjacent molecule at a short distance (3.299–3.473 Å). Furthermore, multiple intermolecular C‐H•••π interactions with close distances (2.533–2.645 Å) are present. These van der Waals forces effectively enhance the intermolecular interactions of M2 in the aggregated state. Since the ISC transition is considered efficient within a small energy gap (Δ*E*<0.3 eV) between S_1_ and T_n_ with sufficient spin‐orbit coupling (SOC), we analyzed the excited singlet and triplet state properties of monomeric, dimeric, and trimeric M2 optimized from the singlet crystal (Figure S30 and Table S6, Supporting Information). As shown in Figure 4B and Table S7 (Supporting Information), monomeric M2 contains two ISC transitions (S_1_→T_1_, T_2_).^[^ ^48^ ^]^ However, dimeric and trimeric M2 exhibit more ISC transition channels (dimer: S_1_→T_1_, T_3_, T_4_, trimer: S_1_→T_3_, T_4_, T_6_) with denser energy level distributions and smaller S_1_‐T_n_ energy gaps compared to monomeric M2 (Figure 4B; Tables S8 and S9, Supporting Information).^[^ ^49^, ^50^ ^]^ The analysis of the electron‐hole distribution reveals different interactions that influence the ISC process. As shown in Figure 4C, the electron‐hole isosurface maps of the T_1_ and T_2_ states of monomeric M2 show that the electron and hole are separately delocalized on the acceptor and donor, respectively, with a large electron‐hole distance (D) (Figures S31, S32 and Table S10, Supporting Information), demonstrating significant intramolecular charge transfer (CT). For dimeric M2, in addition to intramolecular CT (T_1_, T_4_ states), intermolecular CT between the donor of one molecule and the acceptor of another adjacent molecule (T_3_ state) was observed, which effectively facilitates the ISC transition (Figures S33, S34 and Table S11, Supporting Information). The intermolecular CT (T_6_ state) was also observed in trimeric M2 (Figures S35, S36 and Table S12, Supporting Information). Therefore, it is reasonable to infer that as more molecules aggregate, more ISC transitions become accessible due to smaller energy differences and more efficient intramolecular and intermolecular charge transfer. The aggregate‐induced ISC enhancement of M1 and M2 leads to an enhanced Type I ROS generation.

### 2.6 In Vitro and In Vivo PDT Performance Evaluation

**Figure 5:**
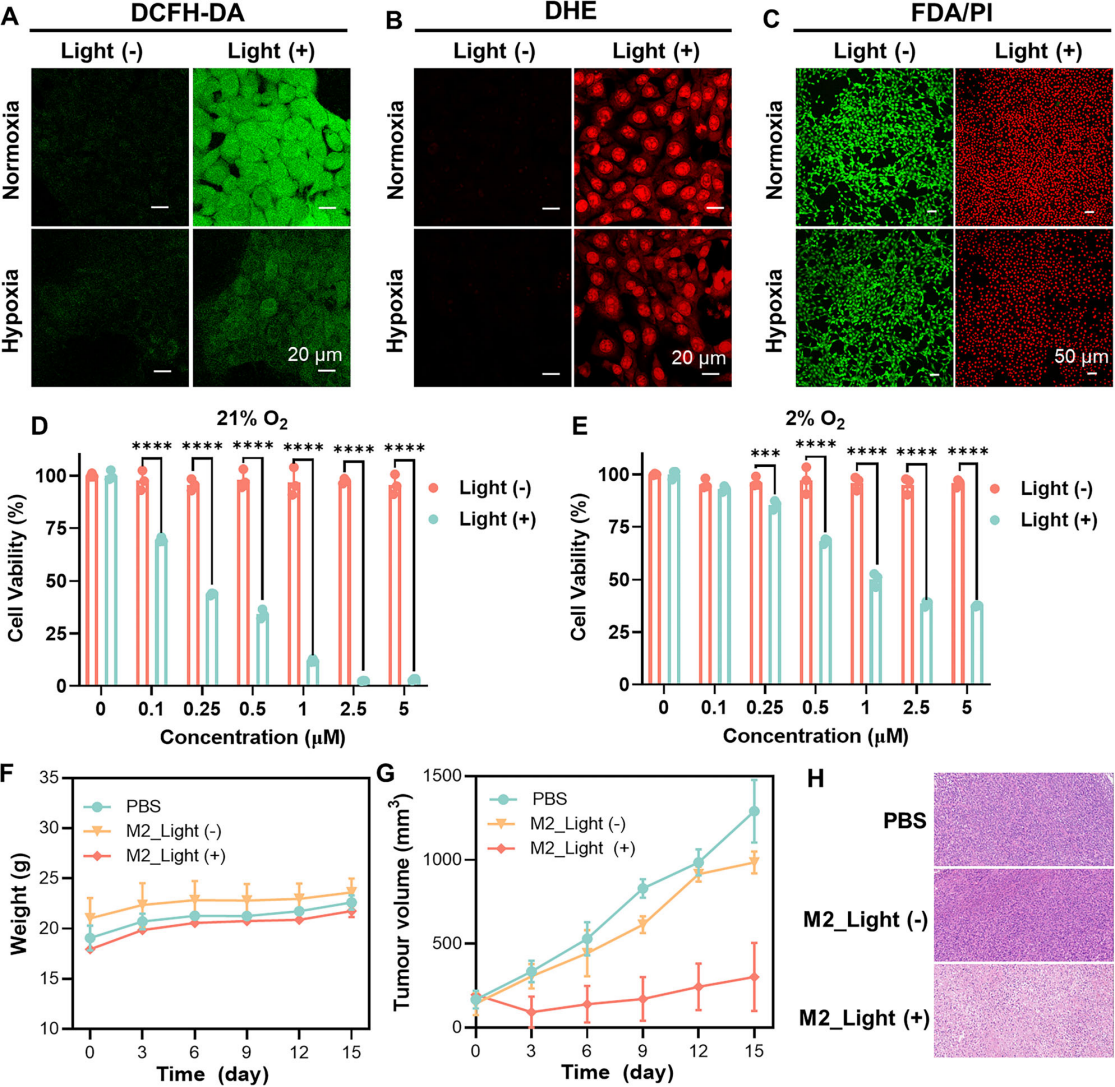
A, B) Fluorescent images of 4T1 cells treated with M2 under white light irradiation in normoxic (21% O_2_) and hypoxic (2% O_2_) conditions to assess intracellular ROS and O_2_^−•^ generation, using DCFH‐DA (A) and DHR123 (B) as indicators, respectively. C) PI/FDA staining of 4T1 cancer cells treated with M2 under white light irradiation (30 mW cm^−2^) in normoxic (21% O_2_) and hypoxic (2% O_2_) conditions. D, E) Cell viability of 4T1 cancer cells treated with M2 under white light irradiation (30 mW cm^−2^) or in the dark in normoxic (21% O_2_) (D) and hypoxic (2% O_2_) (E) conditions. Data are presented as the mean ± SD (n = 3), ^***^ *P*<0.001, ^****^ *P*<0.0001. F) Average weight of mice from control, M2, M2+light groups over 0–15 days. Data are presented as the mean ± SD (n = 3). G) Average tumor volumes of mice from control, M2, M2+light groups over 0–15 days; H) Hematoxylin and eosin (H&E) staining of tumor tissues from control, M2, M2+light groups.

Since M2 exhibits higher O_2_^−•^ generation efficiencies than M1, we evaluated its intracellular ROS generation capacity in 4T1 murine mammary gland cancer cells using the 2’,7’‐dichlorodihydrofluorescein diacetate (DCFH‐DA) staining method in normoxic and hypoxic conditions. The hypoxic environment is constructed in the incubator chamber (MIC‐101, Billups‐Rothenberg) under a mixture gas atmosphere of 93% N_2_, 2% O_2_, and 5% CO_2_. The fluorescence of probe ROS‐ID in 4T1 cancer cells under hypoxic conditions demonstrated that a hypoxic atmosphere was successfully constructed (Figure S37, Supporting Information). As shown in **Figure** **5**A, green fluorescence was detected in 4T1 cancer cells treated with M2 and DCFH‐DA under light irradiation in both normoxic and hypoxic conditions, indicating efficient ROS generation by M2. In contrast, no fluorescence was observed in cells without light irradiation, demonstrating minimal ROS generation in the dark. To assess intracellular O_2_^−•^ generation, we performed a dihydroethidium (DHE) staining assay. As depicted in Figure 5B and Figure S38 (Supporting Information), the red fluorescence of DHE in 4T1 cells treated with M2 increased over time under light irradiation, both in normoxic and hypoxic conditions, indicating intracellular O_2_^−•^ production. The photo‐induced cytotoxicity of M2 was further confirmed using a live/dead cell co‐staining assay, with fluorescein diacetate (FDA, green fluorescence for live cells) and propidium iodide (PI, red fluorescence for dead cells) as probes. As shown in Figure 4, 5 cancer cells treated with M2 in the absence of light irradiation remained alive, as visualized by the intense green fluorescence. In contrast, only the bright red fluorescence of PI was observed upon light irradiation, confirming the pronounced phototoxicity of M2 under both normoxic and hypoxic conditions. The cytotoxicity of M2 was further characterized by the MTT assay. As depicted in Figure 5D,E, M2 exhibited negligible cytotoxicity in normoxic (21% O_2_) and hypoxic (2% O_2_) conditions without irradiation, implying its good biocompatibility at the cellular level. However, upon exposure to white light irradiation (30 mW cm^−2^, 15 min), M2 displayed dose‐dependent phototoxicity against 4T1 cancer cells, with a half‐maximal inhibitory concentration (IC_50_) value of less than 0.25 µm in a normoxic microenvironment. In a hypoxic atmosphere, M2 still exhibited good photocytotoxicity, effectively inhibiting 4T1 cancer cell growth. Encouraged by the promising intracellular PDT efficiency, the in vivo PDT performance of M2 was evaluated in the 4T1 tumor‐bearing mice. The mice inoculated with 4T1 cancer cells were randomly divided into three groups (n = 4): i) control group, injected with PBS, ii) M2 group, injected with M2 (5 mg kg^−1^) without light irradiation, iii) M2+light group, injected with M2 (5 mg kg^−1^), followed by 20 min of white light irradiation (100 mW cm^−2^) 4 h post‐injection (Figure S39, Supporting Information). Throughout the entire therapeutic period, the body weight changes of the mice in all three groups showed similar growth trends (Figure 5F), indicating that the therapeutic regimen did not cause significant adverse effects on the health of the mice. The tumor volume changes reflect the therapeutic effect of photosensitizers. As shown in Figure 5G and Figure S40 (Supporting Information), the tumor volumes of the control and M2 groups gradually increased over time, demonstrating that both the PBS treatment and M2 alone were insufficient to inhibit tumor growth. In contrast, the tumors in the M2+light group displayed minimal growth, reflecting effective tumor growth inhibition due to the PDT effect of M2 under light illumination, attributed to the generation of a substantial amount of O_2_^−•^. After completing the treatment on the 15th day, the mice were sacrificed. The hematoxylin and eosin (H&E) staining assay corroborated the anti‐tumor effect of M2 under white light illumination (Figure 5H). The effective intracellular Type I ROS generation, along with the in vitro and in vivo antitumor efficiency of M2, demonstrated the potential application of ML‐assisted Type I ROS discovery system.

## 3 Conclusion

In conclusion, we successfully developed a closed‐loop hybrid discovery system to efficiently identify and accurately predict Type I PSs for hypoxic tumor therapy. We constructed a comprehensive molecular dataset to identify 664 potential Type I PSs using a support vector machine (SVM) learning algorithm with high accuracy. This approach utilizes the reformulation of acceptors and donors to modulate the excited‐state properties of PSs, obtained through TD‐DFT calculations, enabling reliable and rapid prediction of their Type I properties. A universal platform was developed to accurately identify Type I PSs by analyzing the energy levels of their singlet and triplet excited states, highlighting the practical applicability of our approach. The closed‐loop design, incorporating TD‐DFT calculations, ML screening, and experimental validation, enables efficient candidate discovery with improved reliability in Type I PS development. Then, two PS candidates (M1 and M2) were selected and synthesized based on their structural novelty, ease of synthesis, and small $\Delta E_{\left(S_{1}-T_{n}\right)}$, and large $\Delta E_{\left(T_{1}-S_{0}\right)}$. Different ROS indicators were used to demonstrate that both M1 and M2 effectively produced O_2_^−•^ and HO• without generating ^1^O_2_. Comprehensive studies involving PDT performance characterization, as well as analyses of excited‐state properties, were conducted to unravel the underlying mechanisms. M1 and M2 exhibited prominent aggregate‐induced Type I ROS enhancement due to the increased ISC transitions in the aggregated state, facilitated by synergistic intramolecular and intermolecular charge transfer interactions. Although M1 and M2 have large $\Delta E_{\left(T_{1}-S_{0}\right)}$, they still do not generate ^1^O_2_, reflecting that the matched energy levels between the triplet excited state of PSs and O_2_ also influence the energy transfer pathway. This helps guide the design of Type I PSs, suggesting that molecules with strong D‐A interactions, high T_1_ energy levels, and strong ICT properties are ideal candidates. Subsequently, M2 demonstrated both in vitro and in vivo antitumor effect due to its superior efficiency in generating O_2_^−•^ compared to the commercial photosensitizer RB. Our work highlights the potential of ML in designing high‐performance Type I PSs. Future work will address data scale and biological complexity in Type I photosensitizer discovery. Future work will expand the datasets, synthesize more molecules, and refine closed‐loop optimization strategies for Type I PS discovery.

## 4 Experimental Section

### Collection of Training Dataset

The compounds used for model training were obtained from publications and curated datasets.^[^ ^4^, ^17^, ^30^, ^51^, ^52^, ^53^, ^54^, ^55^, ^56^, ^57^, ^58^, ^59^, ^60^ ^]^ Each molecule was annotated with a binary label, 1 for Type I PSs, generate ROS predominantly via a Type I pathway, and 0 for non–Type I PSs with no Type I behaviors. The final dataset consists of 77 molecules in total, with 40.3% Type I PSs and 59.7% non–Type I PSs. This distribution reflects the natural prevalence of Type I and non–Type I PSs were collected. Including diverse non–Type I samples helps the model learn clear classification boundaries. Although moderately imbalanced, the dataset was handled with data augmentation and cross‐validation. All molecular structures were standardized, and their excited‐state properties were calculated using TD‐DFT to provide consistent and reliable descriptors for model development.

### Construction of a Molecular Set

A specialized generative algorithm was employed to systematically create a complete searching space for photosensitizers, utilizing a predefined list of donor, acceptor, and bridge molecular substructures with fixed bonding positions. The molecular structures, originally in SMILES format, were converted into RDKIT mol object format for further manipulation.^[^ ^61^ ^]^ Donors and acceptors were conjugated fragments that contain rich‐electron and electron‐deficient functional groups, respectively. Donor substructures were assigned to a single bonding site, while acceptors often had multiple connection points. In the construction of DA‐type photosensitizers (donor‐bridge‐acceptor), a bridge was inserted between the donor and acceptor, with each molecule connected at specific pre‐determined positions. For DAD‐type photosensitizers (donor‐bridge‐acceptor‐bridge‐donor), the same bridge was used to link two donors to a central acceptor. Symmetric DAD structures were formed when identical donor molecules were used, whereas asymmetric DADs arise when different donors were connected. This generative process was repeated for all possible combinations derived from the initial substructure list until all DA and DAD photosensitizers were created. Finally, RDKIT was used to validate the chemical structures, and any chemically invalid molecules were excluded from the database and sent for TD‐DFT calculations. This combinatorial generation approach, grounded in chemical fragment‐based design, ensured extensive coverage of the design space while avoiding structurally redundant or chemically implausible candidates.

### Machine Learning Models Analysis

The dataset consists entirely of numerical features, treated as continuous variables, which eliminates the need for any additional encoding processes. Instead of using the molecular fingerprint as the input feature, its associated excited‐state properties were already incorporated within the feature set. For preprocessing, the data were standardized using the StandardScaler from scikit‐learn, ensuring that each feature has a mean of zero and a standard deviation of one, and their performances were compared using cross‐validated accuracy scores. 5 classification algorithms were implemented, including k‐nearest neighbors (KNN), support vector machines (SVM), neural networks (NN), random forest (RF), and extreme gradient boosting (XGB). To fine‐tune the hyperparameters of the non‐linear models, the GridSearchCV function was used, optimizing for accuracy after performing 5 fold cross‐validation, with the dataset split randomly into 80% training and 20% test data. Cross‐validation was employed to estimate the error of the model, providing a more stable assessment of model performance. Among the classifiers, the support vector machine (SVM) algorithm delivered the best results in terms of accuracy. SVM operates by finding the optimal hyperplane that separates the data points into different classes in the feature space. The performance of the algorithm improves as it maximizes the margin between the closest points of the classes, ensuring better generalization on unseen data. After training the model, feature importance was analyzed using SHapley Additive exPlanations (SHAP), providing insights into how each input feature impacts the predictions of the model. SHAP values illustrate the contribution of each feature to the prediction process, allowing for a more interpretable model. The SHAP formula is expressed as:

(1) $$\begin{array}{c} \phi_{i}=\sum \left(w\times \Delta P\right) \end{array}$$

where ϕ_*i*_ is the SHAP value for feature *i*, which quantifies the contribution of feature *i* to the prediction of the model, *w* is the weight assigned to each combination of features, and Δ*P* is the difference in prediction.

### Visualization of the Dataset

In this study, UMAP, a powerful tool for dimensionality reduction, was used to visualize molecular data. 2048‐bit extended connectivity fingerprints (ECFPs) with a radius of 2 were generated using RDKit, applying this uniformly to our dataset. UMAP then condensed these high‐dimensional fingerprints into a 2D space, where it identified nearest neighbors of each molecule to construct a graph, learning the approximate manifold before reducing it to two components. By setting a neighborhood size of 15 and a minimum distance of 0.5 between points, UMAP was optimized to cluster molecules with similar features effectively (Type I and non‐Type I PSs). This approach helped us preserve the high‐dimensional structure, enhancing our analysis of the discovery of Type I photosensitizers.

### Theoretical Computation

The B3LYP hybrid functional coupled with the 6‐31G(d) basis set was employed for the optimization of molecular ground states. Subsequently, the exploration of excited‐state properties was conducted through the time‐dependent density functional theory (TD‐DFT), leveraging the previously optimized ground state geometries as the reference. Gaussian 09 software served as the computational backbone for all quantum chemistry calculations in this work. The spin‐orbital coupling (SOC) matrix elements between singlet and triplet states were calculated using the ORCA program with the B3LYP functional and the 6‐31G(d) basis set. The electron and hole distributions were analyzed on Multiwfn 3.8 software and rendered by VMD 1.9.3.

### Data, Code, and User Interface Availability

All code and data related to preprocessing and classification analysis were hosted on GitHub (https://github.com/zhuyixintc/Type_I_Photosensitizers_Design). Machine learning models were developed using scikit‐learn, while SHAP values for feature importance were generated with the SHAP library in Python programming.

### Synthesis of Compound 3

1,2‐Dicyano‐4‐iodobenzene (1.36 g, 5.35 mmols), 2‐thiopheneboronic acid (2.22 g, 16.0 mmols), K_2_CO_3_ (3.69 g, 26.8 mmols), and [PdCl_2_(dppf)]CH_2_Cl_2_ (388 mg, 0.535 mmols) were dissolved in the mixture solution of tetrahydrofuran/water (THF/H_2_O, 50 mL/10 mL). The reaction mixture was stirred at 90°C in an argon ambient for 18 h. Then the reaction mixture was concentrated in vacuo and added with water (50 mL). It was extracted with dichloromethane (DCM, 50 mL*3 times). The organic phase solution was dried with anhydrous sodium sulfate (Na_2_SO_4_) and concentrated in vacuo to give a residue. The residue was purified via silica gel chromatography (50% DCM in hexane) to afford the product (white solid, 1.1 g, yield 90%). ^1^H NMR (CDCl_3_, 400 MHz): δ 7.99 (d, *J* = 7.2 Hz, 1H), 7.90 (t, *J*_1_ = 15.6 Hz, *J*_2_ = 8.0 Hz, 1H), 7.78 (t, *J*_1_ = 16.4 Hz, *J*_2_ = 8.0 Hz, 1H), 7.50 (t, *J*_1_ = 11.6 Hz, *J*_2_ = 5.2 Hz, 2H), 7.17 (t, *J*_1_ = 7.2 Hz, *J*_2_ = 4.0 Hz, 1H); ^13^C NMR (CDCl_3_, 101 MHz): δ 139.64, 139.54, 134.10, 133.60, 133.12, 130.10, 129.44, 129.06, 128.82, 126.58, 116.74, 115.44; ESI‐MS: m/z 221.0501 [M+].

### Synthesis of Compound 4

Compound 3 (315 mg, 1.5 mmols) and NBS (344 mg, 2 mmols) were dissolved in 1,1,1,3,3,3‐hexafluoro‐2‐propanol (10 mL). The reaction mixture was stirred at room temperature for 6 h. Then the reaction was added with water (30 mL) and extracted with DCM (30 mL*3 times). The organic phase solution was dried with anhydrous Na_2_SO_4_ and concentrated in vacuo to give a residue. The residue was purified via silica gel chromatography (gradient, 0‐50% DCM in hexane) to afford the product (white solid, 383 mg, yield 88%). ^1^H NMR (CDCl_3_, 400 MHz): δ 7.89 (d, *J* = 7.2 Hz, 1H), 7.79 (d, *J* = 7.6 Hz, 2H), 7.25‐7.22 (m, 1H), 7.15‐7.12 (m, 1H); ^13^C NMR (CDCl_3_, 101 MHz): δ 140.83, 138.54, 134.23, 131.89, 129.70, 129.10, 126.81, 116.93, 116.27, 115.28, 115.03, 113.70.

### Synthesis of Compound M1

Compound 4 (90 mg, 0.31 mmols), phenoxazine (55 mg, 0.3 mmols), Pd_2_(dba)_3_ (8 mg, 0.009 mmols), (*t*‐Bu)_3_PHBF_4_ (6 mg, 0.018 mmols), and sodium *tert*‐butoxide (96 mg, 1 mmol) were dissolved in toluene (20 mL). The reaction mixture was stirred at 120°C in an argon atmosphere for 1 h. Then the reaction mixture was added water (30 mL) and extracted with DCM (30 mL*3 times). The organic phase solution was dried with anhydrous Na_2_SO_4_ for 1 h and concentrated in vacuo to give a residue. The residue was purified via silica gel chromatography (gradient, 0‐25% DCM in hexane) to afford the product (red solid, 10 mg, yield 8%). ^1^H NMR (DMSO‐*d*_6_, 400 MHz): δ 8.59 (s, 1H), 8.19 (s, 2H), 8.03 (d, *J* = 3.6 Hz, 1H), 7.40 (d, *J* = 4.0 Hz, 1H), 6.85‐6.81 (m, 6H), 6.37‐6.35 (m, 2H); ^13^C NMR (DMSO‐*d*_6_, 101 MHz): δ 143.66, 142.76, 140.00, 138.71, 135.23, 133.36, 132.02, 130.61, 130.34, 128.12, 124.51, 123.43, 116.48, 116.22, 116.18, 114.74, 113.29; ESI‐HRMS: m/z 391.0773 [M+].

### Synthesis of Compound 5

4‐Bromophthalonitrile (1 g, 4.8 mmols), bis(pinacolato)diboron (1.35 g, 5.3 mmols), KOAc (2.35 g, 24 mmols), and [PdCl_2_(dppf)]CH_2_Cl_2_ (391 mg, 0.48 mmols) were dissolved in 1,4‐dioxane (30 mL). The reaction mixture was stirred at 85°C under an argon atmosphere for 4 h. The reaction was quenched with saturated ammonium chloride (NH_4_Cl) aqueous solution (50 mL) and extracted with ethyl acetate (50 mL*3 times). The organic phase solution was dried with anhydrous Na_2_SO_4_ for 1 h and concentrated in vacuo to give a residue. The residue was purified via silica gel chromatography (gradient, 0‐100% DCM in hexane) to afford the product (white solid, 460 mg, yield 38%). ^1^H NMR (CDCl_3_, 400 MHz): δ 8.21 (s, 1H), 8.11 (d, *J* = 4.0 Hz, 1H), 7.79 (d, *J* = 7.6 Hz, 1H), 1.36 (s, 12H); ^13^C NMR (CDCl_3_, 101 MHz): δ 139.41, 138.81, 132.57, 117.52, 115.47, 115.43, 115.19, 85.26, 24.86; ESI‐MS: m/z 254.1478 [M+].

### Synthesis of Compound 6

2‐Bromothiophene (1452 µL, 15 mmols), 9,9‐dimethyl‐9,10‐dihydro‐acridine (3135 mg, 15 mmols), [PdCl_2_(dppf)]CH_2_Cl_2_ (412 mg, 0.45 mmols), (*t*‐Bu)_3_PHBF_4_ (216 mg, 0.9 mmols), and sodium *tert*‐butoxide (4.32 g, 45 mmols) were dissolved in anhydrous toluene (50 mL). The reaction mixture was stirred at 120°C under argon for 5 h. Then the reaction mixture was added with water (100 mL) and extracted with DCM (100 mL*3 times). The organic phase solution was dried with anhydrous Na_2_SO_4_ for 1 h and concentrated in vacuo to give a residue. The residue was purified via silica gel chromatography (5% DCM in hexane) afforded the product (white solid, 3.5 g, yield 80%). ^1^H NMR (CDCl_3_, 400 MHz): δ 7.44 (t, *J*_1_ = 13.6 Hz, *J*_2_ = 6.0 Hz, 3H), 7.16 (t, *J*_1_ = 8.0 Hz, *J*_2_ = 3.6 Hz, 1H), 7.06 (t, *J*_1_ = 15.6 Hz, *J*_2_ = 7.6 Hz, 2H), 7.03 (s, 1H), 6.97 (t, *J*_1_ = 14.8 Hz, *J*_2_ = 7.2 Hz, 2H), 6.63 (d, *J* = 8.4 Hz, 2H), 1.67 (s, 6H); ^13^C NMR (CDCl_3_, 101 MHz): δ 143.38, 140.96, 130.79, 128.13, 126.61, 126.57, 126.08, 125.20, 121.45, 114.69, 35.89, 31.51; ESI‐MS: m/z 292.1323 [M+].

### Synthesis of Compound 7

Compound 6 (3.5 g, 12 mmols) was dissolved in anhydrous THF (30 mL). The anhydrous THF solution (10 mL) of NBS (2.6 g, 13.8 mmols) was added dropwise into the reaction mixture at 0°C in an argon atmosphere. The reaction mixture was continually stirred for 1 h. Then the reaction was quenched with water (50 mL) and extracted with DCM (50 mL*3 times). The organic phase solution was dried with anhydrous Na_2_SO_4_ for 1 h and concentrated in vacuo to give a residue. The residue was purified via silica gel chromatography (5% DCM in hexane) to obtain the product (white solid, 3.7 g, yield 85%). ^1^H NMR (CDCl_3_, 400 MHz): δ 7.44 (d, *J* = 7.6 Hz, 2H), 7.15 (d, *J* = 3.6 Hz, 1H), 7.08 (t, *J*_1_ = 15.6 Hz, *J*_2_ = 7.6 Hz, 2H), 7.00 (t, *J*_1_ = 14.8 Hz, *J*_2_ = 7.2 Hz, 2H), 6.81 (d, *J* = 8.0 Hz, 1H), 6.70 (d, *J* = 8.0 Hz, 2H), 1.66 (s, 6H); ^13^C NMR (CDCl_3_, 101 MHz): δ 143.94, 140.46, 130.88, 129.19, 126.70, 125.33, 121.85, 114.63, 111.87, 35.84, 31.51.

### Synthesis of Compound M2

Compound 7 (369 mg, 1 mmol), compound 5 (508 mg, 2 mmols), Pd(PPh_3_)_2_Cl_2_ (70 mg, 0.1 mmols), and K_2_CO_3_ (1.38 g, 10 mmols) were dissolved in the THF/H_2_O mixture (40 mL/10 mL). The reaction mixture was stirred at 60°C under argon for 1 h. Then the reaction was quenched with water (50 mL) and extracted with DCM (50 mL*3 times). The organic phase solution was dried with anhydrous Na_2_SO_4_ for 1 h and concentrated in vacuo to give a residue. The residue was purified via silica gel chromatography (50% DCM in hexane) to obtain the product (white solid, 357 mg, yield 72%). ^1^H NMR (CDCl_3_, 400 MHz): δ 8.03 (s, 1H), 7.91 (d, *J* = 8.4 Hz, 1H), 7.82 (d, *J* = 8.0 Hz, 1H), 7.57 (d, *J* = 3.6 Hz, 1H), 7.48 (d, *J* = 7.6 Hz, 2H), 7.12‐7.08 (m, 3H), 7.04 (t, *J*_1_ = 14.8 Hz, *J*_2_ = 7.6 Hz, 2H), 6.73 (d, *J* = 8.0 Hz, 2H), 1.69 (s, 6H); ^13^C NMR (CDCl_3_, 101 MHz): δ 146.92, 140.43, 139.24, 138.78, 134.17, 131.48, 129.93, 129.86, 129.34, 126.72, 125.92, 125.41, 122.22, 116.84, 115.38, 115.15, 114.84, 113.64, 35.98, 31.28; ESI‐HRMS: m/z 418.1336 [M+H].

### Total ROS Detection in Aqueous Solution

The total ROS generation was measured by monitoring the oxidation of DCFH into DCF, accompanied by the appearance of green fluorescence at 525 nm. For the preparation of DCFH solution, 0.5 mL 1 mm DCFH‐DA solution (using ethanol as solvent) was added to 2 mL 10 mm NaOH solution (using water as solvent) to hydrolyze as DCFH for 30 min in the dark. Subsequently, 10 mL 2.5X PBS was added to quench the reaction and acquire the DCFH solution (40 µm), which was batch stored in the dark at −20°C fridge. The mixture of 5 µm DCFH and 10 µm M1/M2/RB in DMSO/PBS solution (v:v = 1:99) was irradiated under white light (30 mW cm^−2^) for 0–3 min, and fluorescent emission spectra between 500–650 nm were measured under 488 nm excitation by using a spectrofluorometer.

### O_2_^−•^ Detection in Aqueous or Organic Solutions

For the detection of O_2_^−•^ in aqueous solution, the mixture of DHR123 (10 µm) and M1/M2/RB (10 µm) in DMSO/H_2_O solution (v:v = 1:99) was irradiated under white light (30 mW cm^−2^) for 0–3 min and fluorescent emission spectra between 500–650 nm were measured upon 488 nm excitation by using spectrofluorometer. For the detection of O_2_^−•^ in organic solution, dissolved DHR123 (10 µm) and M1/M2/RB (10 µm) in the mixture of THF/H_2_O with different water fractions (0%, 25%, 50%, 75% and 99% water). Then the mixture solutions were irradiated under white light (30 mW cm^−2^) for 0 and 3 min. Their fluorescent emission spectra between 500–650 nm were measured upon 488 nm excitation by using a spectrofluorometer.

### HO• Detection in Aqueous Solution

The DMF/PBS (v:v = 1:99) mixture solutions of APF (5 µm) and M1/M2/RB (10 µm) were irradiated under white light (30 mW cm^−2^) for 0–3 min, and fluorescent emission spectra between 500–650 nm were measured upon 488 nm excitation by using a spectrofluorometer.

### ^1^O_2_ Detection in Aqueous or Organic Solutions

For the measurement of ^1^O_2_ in aqueous solution, ABDA and SOSG were used as indicators. The mixture of ABDA (100 µm) and M1/M2/RB (10 µm) in DMSO/H_2_O solution (v:v = 1:99) was irradiated under white light (30 mW cm^−2^) for 0–3 min, and their absorption spectra between 320–420 nm were measured by using a UV–vis spectrometer. The mixture of SOSG (10 µm) and M1/M2/RB (10 µm) in DMSO/H_2_O solution (v:v = 1:99) was irradiated under white light (30 mW cm^−2^) for 0–3 min, and their fluorescent emission spectra between 500–650 nm were measured upon 488 nm excitation by using a spectrofluorometer. For the measurement of ^1^O_2_ in THF, DPBF was utilized as the indicator with the decomposition of DPBF at 415 nm. The THF solution of DPBF (50 µm) and M1/M2/RB (10 µm) was irradiated under white light (30 mW cm^−2^) for 0–30 s, and their absorption spectra between 300–700 nm were measured by using a UV–vis spectrometer.

### Cell Culture

4T1 cells were cultured in DMEM medium with 10% fetal bovine serum (FBS) and 1% penicillin‐streptomycin at 37°C in a humid atmosphere with 5% CO_2_. All the hypoxic experiments were done in the incubator chamber (MIC‐101, Billups‐rothenberg) under a mixture gas atmosphere of 93% N_2_, 2% O_2_, and 5% CO_2_.

### Intracellular ROS Detection

The intracellular ROS generation capacities of M2 were characterized by using DCFH‐DA as an indicator. The 4T1 cells in cell culture were treated with 20 µm M2 for 2 h in hypoxic or normoxic conditions and then treated with 10 µm DCFH‐DA for 30 min. After white light irradiation (30 mW cm^−2^) for 10 min, the fluorescent images of cells were taken using a confocal laser scanning microscope (CLSM). For DCFH channel: λ_ex_ = 488 nm, λ_em_ = 500‐600 nm, power: 10%, gain: 1000.

### Intracellular O_2_^−•^ Detection

The intracellular O_2_^−•^ generation capacities of M2 were characterized by using DHE as the indicator. The 4T1 cells were first treated with 20 µm M2 for 2 h in hypoxic or normoxic conditions. Then the DHE (10 µm) solution was added, and the fluorescence images of cells were taken immediately before or after irradiating for different times using a 405 nm laser. For DHE channel: λ_ex_ = 510 nm, λ_em_ = 550–700 nm, power: 5%, gain: 1000; for irradiation channel: λ_ex_ = 405 nm, power: 0.5%.

### Intracellular Photocytotoxicity Detection

As for the MTT experiment, 4T1 cells were seeded into the 96‐well plate with a density of 1X10^5^ cells/mL. After incubation for 12 h, cell culture medium containing different concentrations of M2 (100 µL) was added to each well. Then cells were further incubated for 4 h in normoxic or hypoxic atmosphere and irradiated under white light (30 mW cm^−2^) for 15 min. After continuing incubating for 8 h, the DMEM solution of MTT (0.5 mg mL^−1^, 100 µL) was added to each well. After incubation at 37°C for 2 h, the MTT solution was removed, and 100 µL DMSO was added to each well, and the plate was shaken for 10 min at room temperature to dissolve all the precipitates. The absorbance of the solution at 570 nm was measured by a microplate reader. Cell viability was calculated by the ratio of the absorbance of the sample and the control group. About the live/dead staining experiment, the 4T1 cells were seeded into the glass‐bottom dishes at a density of 1X10^5^ cells/mL. The 4T1 cells in cell culture medium were first treated with 20 µM M2 for 4 h in hypoxic or normoxic ambient and then irradiated under white light (30 mW/cm^2^) for 15 min. After continuing incubation for 4 h, cells were incubated with 30 µg mL^−1^ PI and 10 µg mL^−1^ FDA for 40 min. Then the fluorescent images of cells were taken. For PI channel: λ_ex_ = 520 nm, λ_em_ = 600–640 nm, power: 5%, gain: 1000; for FDA channel: λ_ex_ = 470 nm, λ_em_ = 500–540 nm, power: 2%, gain: 800.

### In Vivo Tumorous Inhibition Experiment

Each animal's produces was approved by the Institutional Animal Care and Use Committee (IACUC), the First Affiliated Hospital of Sun Yat‐Sen University (NO. [2023]311). To build the tumor‐bearing mice, 100 µL of PBS with 1 × 10^6^ 4T1 cancer cells was injected into the axilla of BALB/c female mice. When the tumors grew to ≈150 mm^3^, the mice were randomly divided into 3 groups with 4 mice in each group: i) control group, injected with PBS, ii) M2 group, injected with M2 (5 mg kg^−1^) without light irradiation, iii) M2 + light group, injected with M2 (5 mg kg^−1^) with light irradiation. Animals were treated via in situ injection. The tumor site was irradiated under white light for 20 min (100 mW cm^−2^) at 4 h post‐injection. Tumor volumes and body weights were measured every three days. Tumor volumes were obtained by measuring the perpendicular diameter of the tumor in length and width and calculated according to the formula: Tumor volume (mm^3^) = 1/2 × length × width^2^. At the last time point, the mice were sacrificed, and the tumor tissues were collected for H&E staining.

### Statistical Analysis

Each experiment was performed in triplicate, with results expressed as mean ± SD. Statistical analysis was carried out using GraphPad Prism 9 software. Intergroup differences were evaluated by a two‐tailed Student's *t*‐test, and statistical significance was set at ^***^ *P*<0.001, ^****^ *P*<0.0001. All figures were plotted with Origin 8.5 software and GraphPad Prism 9.

## Conflict of Interest

The authors declare no conflict of interest.

## Supporting information



Supporting Information

Supporting Data

## Data Availability

The data that support the findings of this study are available in the supplementary material of this article.
