# Supplementary material for: A Closed‐Loop Hybrid Discovery System of Type I Photosensitizers for Hypoxic Tumor Therapy
Source: Adv Sci (Weinh). 2025 Dec 12;13(9):e15103. doi: 10.1002/advs.202515103 (PMC12903996; doi:10.1002/advs.202515103)
Supplement: Supplementary file 1 — Supporting Information [file ADVS-13-e15103-s001.docx]

**Supporting Information**

**A Closed-Loop Hybrid Discovery System of Type I Photosensitizers for Hypoxic Tumor Therapy**

Xia Ling,^1,2‡^ Yixin Zhu,^2‡^ Min Li,^3^ Zongliang Xie,^2^ Lei Cao,^1,2^ Wentao Song,^1,2^ Dandan Wang,^1,2^ Duo Mao,^3^ Xiaonan Wang,^4*^ Bin Liu^1,2*^

^1^Joint School of National University of Singapore and Tianjin University, International Campus of Tianjin University, Binhai New City, Fuzhou 350207, China.

^2^Department of Chemical and Biomolecular Engineering, National University of Singapore, Singapore 117585, Singapore.

^3^Institute of Precision Medicine, The First Affiliated Hospital of Sun Yat-Sen University, Sun Yat-Sen University, Guangzhou 510080, China.

^4^Department of Chemical Engineering, Tsinghua University, Beijing 100084, China.

^‡^These authors contributed equally to this work.

^*^Correspondence and requests for materials should be addressed to Xiaonan Wang (email: [wangxiaonan@tsinghua.edu.cn](mailto:wangxiaonan@tsinghua.edu.cn)); Bin Liu (email: [cheliub@nus.edu.sg](mailto:cheliub@nus.edu.sg))

**Contents**

[**Experimental procedures** 4](#_Toc214610832)

[Instruments and materials 4](#_Toc214610833)

[Synthesis and characterization 4](#_Toc214610834)

[**Scheme S1**. Overall synthetic routes of compounds 1-7 5](#_Toc214610835)

[**Supplementary Figures** 6](#_Toc214610836)

[**Figure S1.** SHAP analysis results of excited-state properties of candidates 6](#_Toc214610837)

[**Figure S2.** Interface designed for Type I photosensitizers identification 6](#_Toc214610838)

[**Figure S3.** ^1^H and ^13^C NMR spectra of compound 3 7](#_Toc214610839)

[**Figure S4.** ^1^H and ^13^C NMR spectra of compound 4 8](#_Toc214610840)

[**Figure S5.** ^1^H and ^13^C NMR spectra of M1 9](#_Toc214610841)

[**Figure S6.** ^1^H and ^13^C NMR spectra of compound 5 10](#_Toc214610842)

[**Figure S7.** ^1^H and ^13^C NMR spectra of compound 6 11](#_Toc214610843)

[**Figure S8.** ^1^H and ^13^C NMR spectra of compound 7 12](#_Toc214610844)

[**Figure S9.** ^1^H and ^13^C NMR spectra of M2 13](#_Toc214610845)

[**Figure S10.** UV-Vis absorption and emission spectra of M1 and M2 14](#_Toc214610846)

[**Figure S11.** Aggregate-induced emission behavior of M1 14](#_Toc214610847)

[**Figure S12.** Aggregate-induced emission behavior of M2 15](#_Toc214610848)

[**Figure S13.** O_2_^‒•^ generation capacities of M1/M2 in DCM 15](#_Toc214610849)

[**Figure S14.** ^1^O_2_ generation capacities of M1/M2/RB in DCM 16](#_Toc214610850)

[**Figure S15.** Relative absorption decomposition of DPBF with M1/M2/RB in DCM 16](#_Toc214610851)

[**Figure S16.** Dynamic light scattering (DLS) spectra of M1/M2 in aqueous solution 17](#_Toc214610852)

[**Figure S17.** ROS generation capacities of M1/M2/RB in aqueous solution 17](#_Toc214610853)

[**Figure S18.** O_2_^‒•^ generation capacities of M1/M2/RB in aqueous solution 18](#_Toc214610854)

[**Figure S19.** HO• generation capacities of M1/M2/RB in aqueous solution 19](#_Toc214610855)

[**Figure S20.** ^1^O_2_ generation capacities of M1/M2/RB by using ABDA as indicator 20](#_Toc214610856)

[**Figure S21.** ^1^O_2_ generation capacities of M1/M2/RB by using SOSG as indicator 21](#_Toc214610857)

[**Figure S22.** Relative fluorescence changes of SOSG with M1, M2 and RB in aqueous solution 21](#_Toc214610858)

[**Figure S23.** O_2_^–•^ generation of M1/M2/CV in aqueous solution by using DHR123 as indicator 22](#_Toc214610859)

[**Figure S24**. HO• generation of M1/M2/CV in aqueous solution by using APF as indicators 23](#_Toc214610860)

[**Figure S25**. Relative fluorescence changes of DHR123 and APF with M1, M2 and CV in aqueous solution 23](#_Toc214610861)

[**Figure S26.** O_2_^‒•^ generation capacities of RB in THF/H_2_O mixed solvent 24](#_Toc214610862)

[**Figure S27.** O_2_^‒•^ generation capacities of M1 in THF/H_2_O mixed solvent 24](#_Toc214610863)

[**Figure S28.** O_2_^‒•^ generation capacities of M2 in THF/H_2_O mixed solvent 25](#_Toc214610864)

[**Figure S29.** Packing structures of M2 from its single crystal 25](#_Toc214610865)

[**Figure S30.** Structures of monomer, dimer and trimer of M2 26](#_Toc214610866)

[**Figure S31.** TD-DFT calculation results of singlet excited state of monomeric M2 27](#_Toc214610867)

[**Figure S32.** TD-DFT calculation results of triplet excited state of monomeric M2 27](#_Toc214610868)

[**Figure S33.** TD-DFT calculation results of singlet excited state of dimeric M2 28](#_Toc214610869)

[**Figure S34.** TD-DFT calculation results of triplet excited state of dimeric M2 28](#_Toc214610870)

[**Figure S35.** TD-DFT calculation results of singlet excited state of trimeric M2 29](#_Toc214610871)

[**Figure S36.** TD-DFT calculation results of triplet excited state of trimeric M2 29](#_Toc214610872)

[**Figure S37.** Images of ROS-ID probe in 4T1 cancer cells under hypoxic and normoxic conditions 30](#_Toc214610873)

[**Figure S38.** Intracellular O_2_^‒•^ generation induced by M2 30](#_Toc214610874)

[**Figure S39.** Workflow of the in vivo anti-tumor experiment 30](#_Toc214610875)

[**Figure S40**. Photograph of dissected tumor tissues after 15-day treatment 31](#_Toc214610876)

[**Supplementary Tables** 32](#_Toc214610877)

[**Table S1.** Hyperparameters for the five supervised-learning models employed 32](#_Toc214610878)

[**Table S2.** Parameter settings of the UMAP algorithm used to project high dimension feature space into two dimensions for visualization 32](#_Toc214610879)

[**Table S3.** Vertical excitation energies (eV) of S_1_-S_10_, T_1_-T_10_ excited states of M1 and M2 33](#_Toc214610880)

[**Table S4.** HOMO-LUMO and Gibbs free energy calculation results of M1 and M2 at the B3LYP/6-31G(d) Opt level 34](#_Toc214610881)

[**Table S5.** Crystal data and structure refinement for M2 single crystal^[5]^ 35](#_Toc214610882)

[**Table S6.** Vertical excitation energies (eV) of excited singlet state (S_1_-S_10_), and excited triplet state (T_1_-T_10_) of monomer, dimer and trimer of M2 optimized from M2 single crystal 35](#_Toc214610883)

[**Table S7.** Energy gaps and spin-orbit coupling (SOC) constants of monomeric M2 at S_1_ → T_1_, T_2_ transitions 36](#_Toc214610884)

[**Table S8.** Energy gaps and spin-orbit coupling (SOC) constants of dimeric M2 at S_1_ → T_1_, T_2_, T_3_, T_4_ transitions 36](#_Toc214610885)

[**Table S9.** Energy gaps and spin-orbit coupling (SOC) constants of trimeric M2 at S_1_ → T_1_, T_2_, T_3_, T_4_, T_5_, T_6_, T_7_ transitions 36](#_Toc214610886)

[**Table S10.** TD-DFT calculation results of excited states of monomeric M2 37](#_Toc214610887)

[**Table S11.** TD-DFT calculation results of excited states of dimeric M2 37](#_Toc214610888)

[**Table S12.** TD-DFT calculation results of excited states of trimeric M2 38](#_Toc214610889)

[**Supplementary References** 39](#_Toc214610890)

# **Experimental procedures**

## **Instruments and materials**

All nuclear magnetic resonance (NMR) spectra (^1^H NMR and ^13^C NMR) were tested on a Bruker ARX 400 NMR spectrometer with TMS as the internal reference. High-resolution mass spectra (HRMS) with EI ionization were recorded on an Agilent 7200 GC-QTOF. UV-vis spectra were carried out on a Shimadzu UV-2600 spectrophotometer (Japan). Fluorescent emission spectra were acquired using an FLS5 steady-state spectrometer (Edinburgh). Photoluminescence (PL) spectra were measured on an Ocean Optic QE 65 Pro spectrometer with a reflection probe R600-125F. Single-crystal X-ray analyses were done using a Bruker D8 Venture Single-Crystal X-ray Diffractometer. For MTT assays, the BioTech Synergy H4 microplate reader was used for the absorbance measurement. Confocal images were obtained by Leica SP8 confocal laser scanning microscope (CLSM).

All chemicals and solvents were purchased from commercial sources without further purification. 1,1'-Bis(diphenylphosphino)ferrocene-palladium(II)dichloride dichloromethane complex ([PdCl_2_(dppf)]CH_2_Cl_2_), tris(dibenzylideneacetone)dipalladium (Pd_2_(dba)_3_), sodium sulfate anhydrous (Na_2_SO_4_), potassium acetate (KOAc), ammonium chloride (NH_4_Cl), dimethyl sulfoxide (DMSO), 1,4-dioxane, chloroform-*d* (CDCl_3_), dimethyl sulfoxide-*d*_6_ (DMSO-*d*_6_), 2′,7′-dichlorofluorescin diacetate (DCFH-DA), dihydrorhodamine 123 (DHR123), 9,10-anthracenediyl-bis(methylene)dimalonic acid (ABDA), 3'-(4-aminophenoxy)-6'-hydroxyspiro[2-benzofuran-3,9'-xanthene]-1-one (APF), rose bengal (RB), 1,3-diphenylisobenzofuran (DPBF), hydroethidine (DHE), 3-(4,5-dimethylthiazol-2-yl)-2,5-diphenyltetrazolium bromide (MTT), fluorescein diacetate (FDA), and phosphate-buffered saline (PBS) were purchased from Sigma-Aldrich Co., Ltd. (Singapore), Potassium carbonate (K_2_CO_3_), N-bromosuccinimide (NBS), sodium *tert*-butoxide, 2-bromothiophene, and bis(triphenylphosphine)palladium(II) chloride (Pd(PPh_3_)_2_Cl_2_) were purchased from the Tokyo Chemical Industry Co., Ltd. (Japan). 1,2-Dicyano-4-iodobenzene, 1,1,1,3,3,3-hexafluoro-2-propanol, phenoxazine, tri-*tert*-butylphosphine tetrafluoroborate ((*t*-Bu)_3_PHBF_4_), 4-bromophthalonitrile, and 9,9-dimethyl-9,10-dihydro-acridine were purchased from the BLD Pharmatech Ltd. (China). 2-Thiopheneboronic acid and bis(pinacolato)diboron were purchased from Combi-Blocks company. Tetrahydrofuran (THF), toluene, dichloromethane (DCM), hexane, ethyl acetate (EA), and N, N-dimethylformamide (DMF) were purchased from Fisher Chemical. Singlet oxygen sensor green (SOSG), propidium iodide (PI) and Dulbecco's modified eagle medium (DMEM) were purchased from Thermo Fisher Scientific company.

## **Synthesis and characterization**

All solvents and reagents were used as obtained without further purification, unless otherwise stated. All reactions that required anhydrous conditions were carried out under argon atmosphere using oven-dried glassware. The heating of reactions was accomplished with a silicon oil bath on top of a stirring hotplate equipped with an electronic contact thermometer to maintain the indicated temperature. The reaction process was monitored by analytical thin layer chromatography (TLC) on pre-coated silica plates and spots were visualized by UV (254/365 nm). Flash column chromatography was carried out using 300-400 mesh silica gel. All NMR spectra (^1^H NMR and ^13^C NMR spectra) were collected on a Bruker ARX 400 NMR spectrometer. Chemical shifts (*δ*) are reported in ppm (CDCl_3_ = 7.26 ppm for ^1^H NMR; CDCl_3_ = 77.0 ppm for ^13^C NMR, DMSO-*d*_6_ = 2.50 ppm for ^1^H NMR; DMSO-*d*_6_ = 40.0 ppm for ^13^C NMR). The following abbreviations were used for reporting ^1^H NMR spectra: chemical shift (𝛿 ppm), s = singlet, d = doublet, t = triplet, and m = multiplet. Coupling constants, *J*, are measured to the nearest 0.1 Hz. All the measurements were performed at 25 °C.

## **Scheme S1**. Overall synthetic routes of compounds 1-7

Reagents and conditions: (a) potassium carbonate (K_2_CO_3_), 1,1'-bis(diphenylphosphino)ferrocene-palladium(II)dichloride dichloromethane complex ([PdCl_2_(dppf)]CH_2_Cl_2_), tetrahydrofuran/water (THF/H_2_O), 90℃, argon ambient, 18 h; (b) N-bromosuccinimide (NBS), 1,1,1,3,3,3-hexafluoro-2-propanol, room temperature, argon ambient, 6 h; (c) phenoxazine, tris(dibenzylideneacetone)dipalladium (Pd_2_(dba)_3_), tri-*tert*-butylphosphine tetrafluoroborate ((*t*-Bu)_3_PHBF_4_), sodium *tert*-butoxide, toluene, 120℃, argon ambient, 1 h; (d) potassium acetate (KOAc), [PdCl_2_(dppf)]CH_2_Cl_2,_ 1,4-dioxane, 80℃, 4 h; (e) [PdCl_2_(dppf)]CH_2_Cl_2_, (*t*-Bu)_3_PHBF_4_, sodium *tert*-butoxide, toluene, 120℃, argon ambient, 5 h; (f) NBS, THF, 0℃, argon ambient, 1 h; (g) bis(triphenylphosphine)palladium(II) chloride (Pd(PPh_3_)_2_Cl_2_), (*t*-Bu)_3_PHBF_4_, C_4_H_9_ONa, toluene, 60℃, argon ambient, 1 h.

# **Supplementary Figures**

## **Figure S1.** SHAP analysis results of excited-state properties of candidates

In the bar plot (A), the significance of each of the excited-state properties is illustrated through their SHAP values. A higher SHAP value indicates a more substantial contribution to the predictive ability of the model. Conversely, the dot plot (B) showcases each molecule as a dot, arranged vertically to show density distribution. The values of properties are color-coded: yellow for higher values and purple for lower ones. The SHAP values, plotted on the X-axis, positively correlate with the likelihood of Type I photosensitizer occurrence: a positive SHAP value suggests a higher probability of being Type I photosensitizer.

## **Figure S2.** Interface designed for Type I photosensitizers identification

The interface is ready to start with a progress bar at full completion, awaiting user interaction. By clicking 'Start', users can upload excited-state properties of molecules, after which the software will analyze and output a file classifying the molecules as Type I photosensitizers or not.

## **Figure S3.** ^1^H and ^13^C NMR spectra of compound 3

## **Figure S4.** ^1^H and ^13^C NMR spectra of compound 4

## **Figure S5.** ^1^H and ^13^C NMR spectra of M1

## **Figure S6.** ^1^H and ^13^C NMR spectra of compound 5

## **Figure S7.** ^1^H and ^13^C NMR spectra of compound 6

## **Figure S8.** ^1^H and ^13^C NMR spectra of compound 7

## **Figure S9.** ^1^H and ^13^C NMR spectra of M2

## **Figure S10.** UV-Vis absorption and emission spectra of M1 and M2

Black solid lines represent the UV-Vis absorption spectra of M1 (A) and M2 (B) in DMSO. Red solid lines represent the emission spectra of M1 (A) and M2 (B) in the DMSO/H_2_O mixture (v:v = 1:99), respectively. [M1] = [M2] = 50 μM, λ_ex_ = 405 nm (A) and 390 nm (B).

## **Figure S11.** Aggregate-induced emission behavior of M1

The emission spectra (A) and plot of the fluorescent intensity changes (B) of M1 in THF/H_2_O mixture with different water fractions (*f*_water_, %). [M1] = 10 μM. λ_ex_ = 405 nm.

## **Figure S12.** Aggregate-induced emission behavior of M2

The emission spectra (A) and plot of fluorescent intensity changes (B) of M2 in THF/H_2_O mixture with different water fractions (*f*_water_, %). [M2] = 10 μM. λ_ex_ = 390 nm.

**Figure S13.** O_2_^‒•^ generation capacities of M1/M2 in DCM

The emission spectra and relative changes of fluorescent intensity of DHR123 with M1/M2 in DCM under white light irradiation for 0-3 min. [M1] = [M2] = [DHR123] = 10 μM. λ_ex_= 488 nm.

## **Figure S14.** ^1^O_2_ generation capacities of M1/M2/RB in DCM

The UV-Vis absorption spectra of DPBF in the presence of M1/M2/RB in DCM under white light irradiation for 0-30 s. [M1] = [M2] = [RB] = [10 μM], [DPBF] = 100 μM.

## **Figure S15.** Relative absorption decomposition of DPBF with M1/M2/RB in DCM

.

## **Figure S16.** Dynamic light scattering (DLS) spectra of M1/M2 in aqueous solution

**Figure S17.** ROS generation capacities of M1/M2/RB in aqueous solution

The emission spectra of DCFH in the presence of M1/M2/RB in the solution of PBS/DMSO (v:v = 99:1) under white light irradiation for 0-3 min. [M1] = [M2] = [RB] = 10 μM, [DCFH] = 5 μM. λ_ex_ = 488 nm.

**Figure S18.** O_2_^‒•^ generation capacities of M1/M2/RB in aqueous solution

The emission spectra of DHR123 in the presence of M1/M2/RB in the solution of H_2_O/DMSO (v:v = 99:1) under white light irradiation for 0-3 min. [M1] = [M2] = [RB] = [DHR123] = 10 μM. λ_ex_= 488 nm.

**Figure S19.** HO• generation capacities of M1/M2/RB in aqueous solution

The emission spectra of APF in the presence of M1/M2/RB in the solution of PBS/DMF (v:v = 99:1) under white light irradiation for 0-3 min. [M1] = [M2] = [RB] = 10 μM, [APF] = 5 μM, λ_ex_ = 488 nm.

**Figure S20.** ^1^O_2_ generation capacities of M1/M2/RB by using ABDA as indicator

The absorption spectra of ABDA in the presence of M1/M2/RB in the solution of H_2_O/DMSO (v:v = 99:1) under white light irradiation for 0-3 min. [M1] = [M2] =[RB] =10 μM, [ABDA] = 100 μM.

## **Figure S21.** ^1^O_2_ generation capacities of M1/M2/RB by using SOSG as indicator

The emission spectra of SOSG in the presence of M1/M2/RB in the solution of H_2_O/DMSO (v:v = 99:1) under white light irradiation for different time. [M1] = [M2] = [RB] = [SOSG] = 10 μM, λ_ex_ = 488 nm.

## **Figure S22.** Relative fluorescence changes of SOSG with M1, M2 and RB in aqueous solution

## **Figure S23.** O_2_^–•^ generation of M1/M2/CV in aqueous solution by using DHR123 as indicator

The fluorescence of DHR123 with M1, M2 and CV in aqueous solution under white light irradiation for 3 min. [M1] = [M2] = [CV] = [DHR123] = 10 μM, λ_ex_ = 488 nm.

## **Figure S24**. HO• generation of M1/M2/CV in aqueous solution by using APF as indicators

The fluorescence of APF with M1, M2 and CV in aqueous solution under white light irradiation for 3 min. [APF] = 5 μM, [M1] = [M2] = [CV] = 10 μM. λ_ex_ = 488 nm.

## **Figure S25**. Relative fluorescence changes of DHR123 and APF with M1, M2 and CV in aqueous solution

## **Figure S26.** O_2_^‒•^ generation capacities of RB in THF/H_2_O mixed solvent

The emission spectra of DHR123 in the presence of RB in the mixed solvent of THF/water with different water fractions under white light irradiation for 0-3 min. [RB] = [DHR123] = [10 μM], λ_ex_ = 488 nm. Because indicators have different sensitivities towards ROS in different solvents, RB was utilized as a positive control group to investigate that DHR123 can effectively work in THF and THF/water mixed solvent.

## **Figure S27.** O_2_^‒•^ generation capacities of M1 in THF/H_2_O mixed solvent

The emission spectra of DHR123 in the presence of M1 in the mixed solvent of THF/water with different water fractions under white light irradiation for 0-3 min was collected. [M1] = [DHR123] = [10 μM], λ_ex_ = 488 nm.

## **Figure S28.** O_2_^‒•^ generation capacities of M2 in THF/H_2_O mixed solvent

The emission spectra of DHR123 in the presence of M2 in the mixture solvent of THF/water with different water fractions under white light irradiation for 0-3 min was collected. [M2] = [DHR123] = [10 μM], λ_ex_ = 488 nm.

## **Figure S29.** Packing structures of M2 from its single crystal

There are multiple intermolecular interactions of M2 from its single crystal. The acceptor of M2 forms π-π stacking with the donor of adjacent M2 molecule with a close distance (3.299-3.473 Å). The acceptor of M2 has a C-H•••π interaction with the donor of adjacent M2 molecule with a close distance (2.533 Å). The thiophene π-bridge forms C-H•••π interaction with the donor of adjacent molecule with a close distance (2.645 Å).

## **Figure S30.** Structures of monomer, dimer and trimer of M2

These structures were extracted from the single crystal of M2 for TD-DFT calculations. Green, red and blue lines represent different monomers in each subfigure.


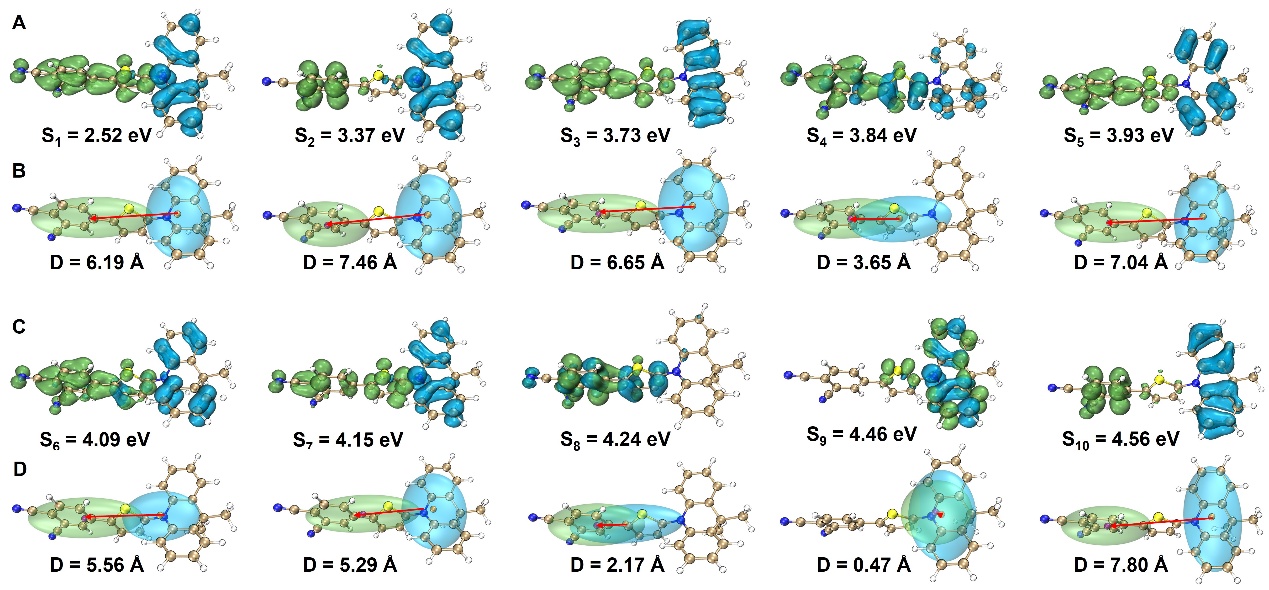


## **Figure S31.** TD-DFT calculation results of singlet excited state of monomeric M2

The isosurface maps of electron-hole distribution (A, C) and Cele-Chole distribution (B, D) of monomeric M2 at singlet excited state (S_1_-S_10_). Bule and green isosurfaces correspond to hole and electron distribution, respectively. Chole and Cele are the smooth distribution of holes (blue regions) and electrons (green regions). The orange ball and purple ball represent the hole and electron center of mass, respectively. The red arrows represent charge transfer from the hole to the electron center of mass. D is the distance between the hole and electron center of mass.


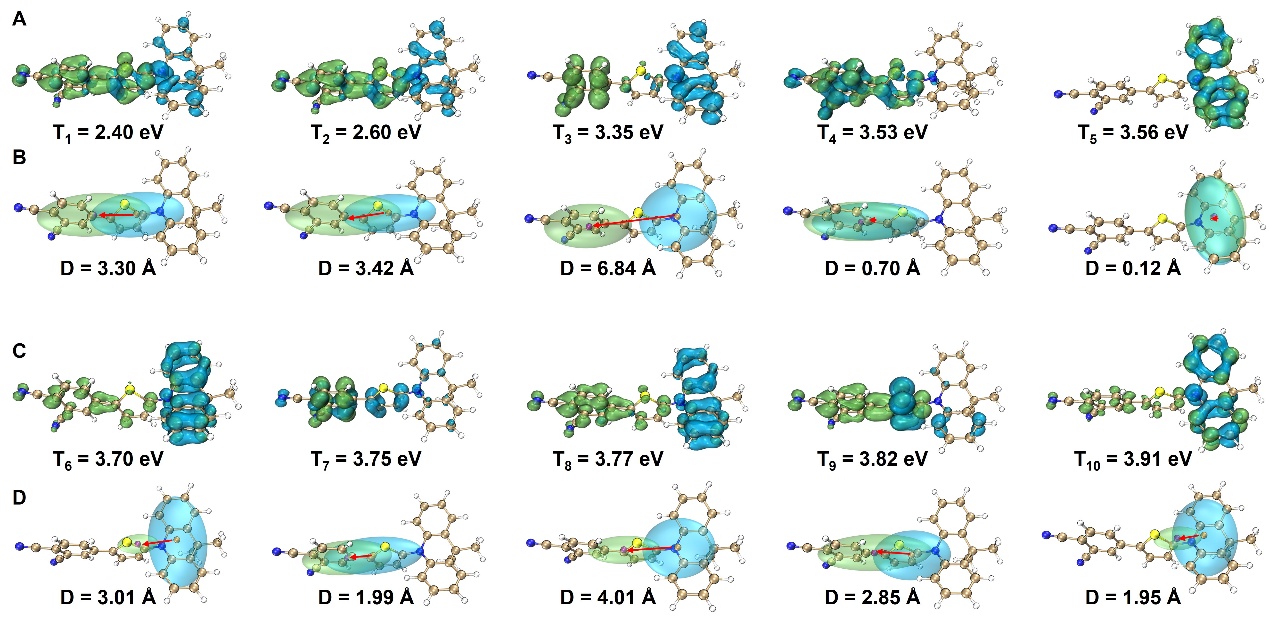


## **Figure S32.** TD-DFT calculation results of triplet excited state of monomeric M2

The isosurface maps of electron-hole distribution (A, C) and Cele-Chole distribution (B, D) of monomeric M2 at triplet excited state (T_1_-T_10_). Bule and green isosurfaces correspond to hole and electron distribution, respectively. Chole and Cele are the smooth distribution of holes (blue regions) and electrons (green regions). The orange ball and purple ball represent the hole and electron center of mass, respectively. The red arrows represent charge transfer from the hole to the electron center of mass. D is the distance between the hole and electron center of mass.


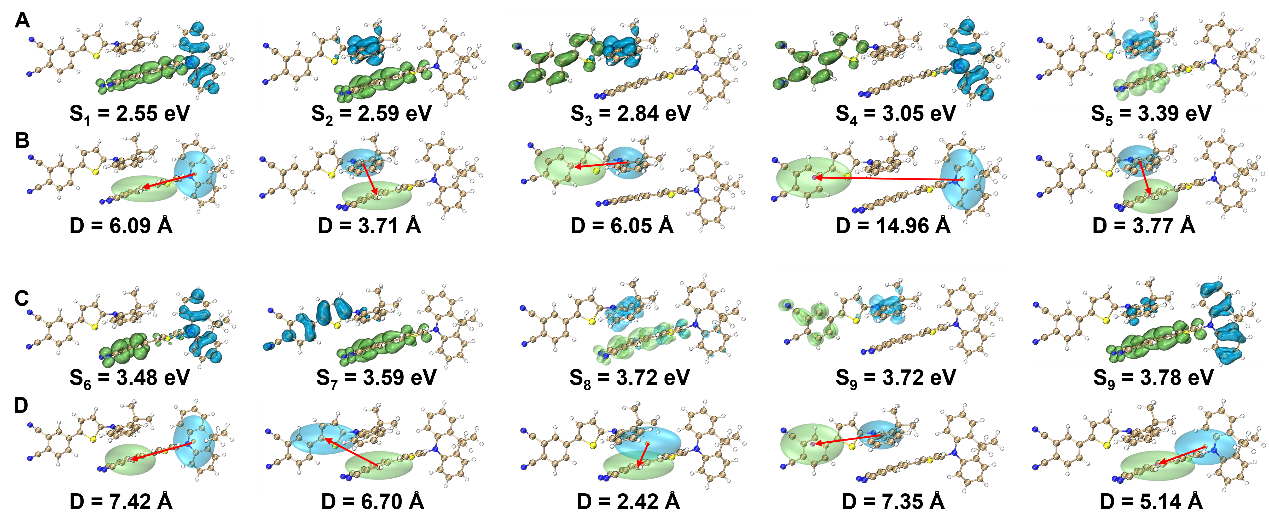


## **Figure S33.** TD-DFT calculation results of singlet excited state of dimeric M2

The isosurface maps of electron-hole distribution (A, C) and Cele-Chole distribution (B, D) of dimeric M2 at singlet excited state (S_1_-S_10_). Bule and green isosurfaces correspond to hole and electron distribution, respectively. Chole and Cele are the smooth distribution of holes (blue regions) and electrons (green regions). The orange ball and purple ball represent the hole and electron center of mass, respectively. The red arrows represent charge transfer from the hole to the electron center of mass. D is the distance between the hole and electron center of mass.


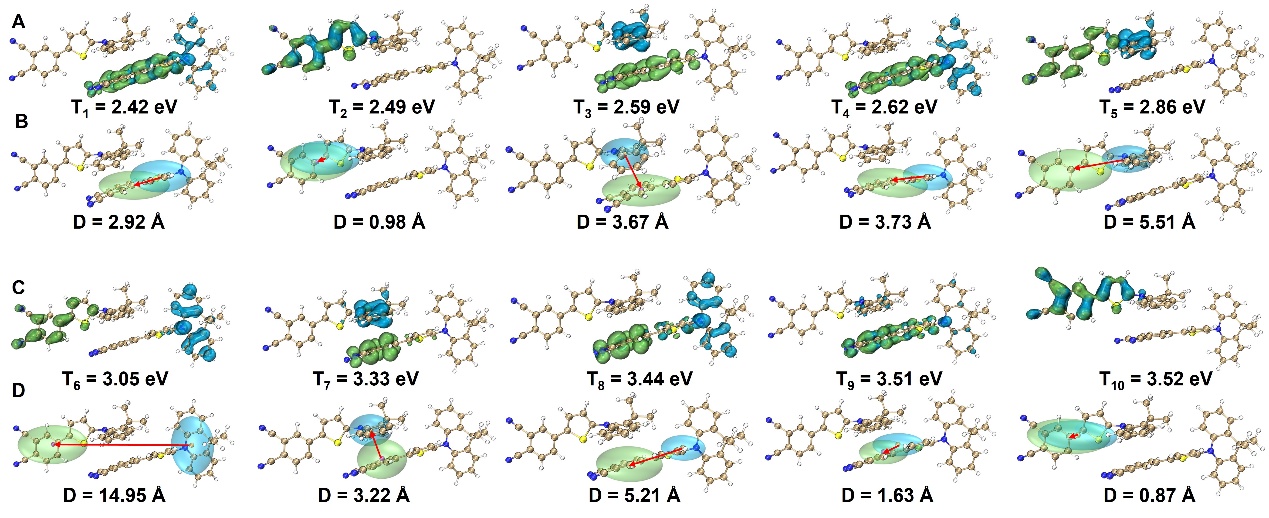


## **Figure S34.** TD-DFT calculation results of triplet excited state of dimeric M2

The isosurface maps of electron-hole distribution (A, C) and Cele-Chole distribution (B, D) of dimeric M2 at triplet excited state (T_1_-T_10_). Bule and green isosurfaces correspond to hole and electron distribution, respectively. Chole and Cele are the smooth distribution of holes (blue regions) and electrons (green regions). The orange ball and purple ball represent the hole and electron center of mass, respectively. The red arrows represent charge transfer from the hole to the electron center of mass. D is the distance between the hole and electron center of mass.


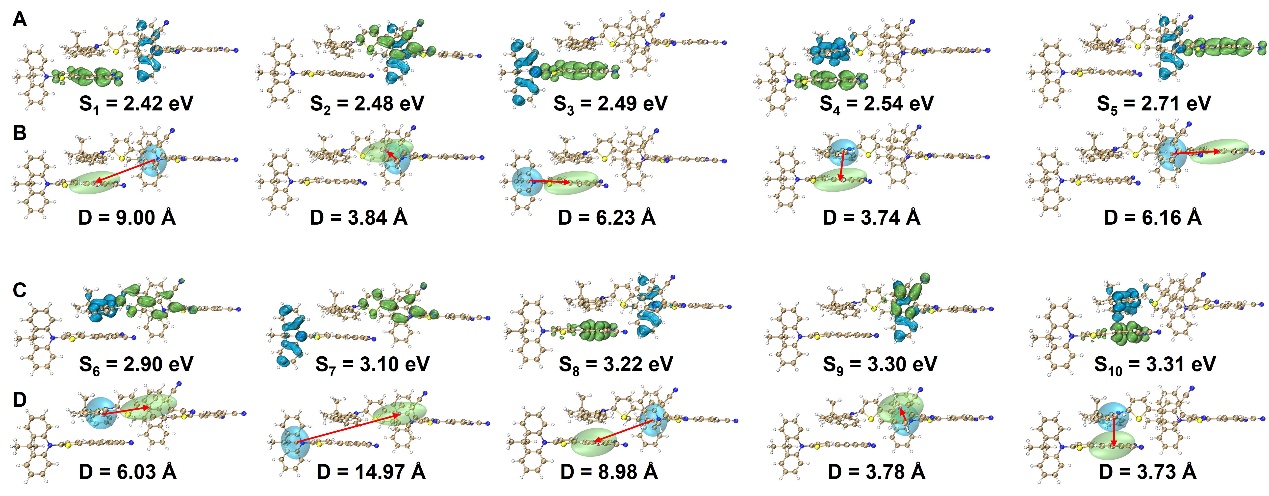


## **Figure S35.** TD-DFT calculation results of singlet excited state of trimeric M2

The isosurface maps of electron-hole distribution (A, C) and Cele-Chole distribution (B, D) of trimeric M2 at singlet excited state (S_1_-S_10_). Bule and green isosurfaces correspond to hole and electron distribution, respectively. Chole and Cele are the smooth distribution of holes (blue regions) and electrons (green regions). The orange ball and purple ball represent the hole and electron center of mass, respectively. The red arrows represent charge transfer from the hole to the electron center of mass. D is the distance between the hole and electron center of mass.


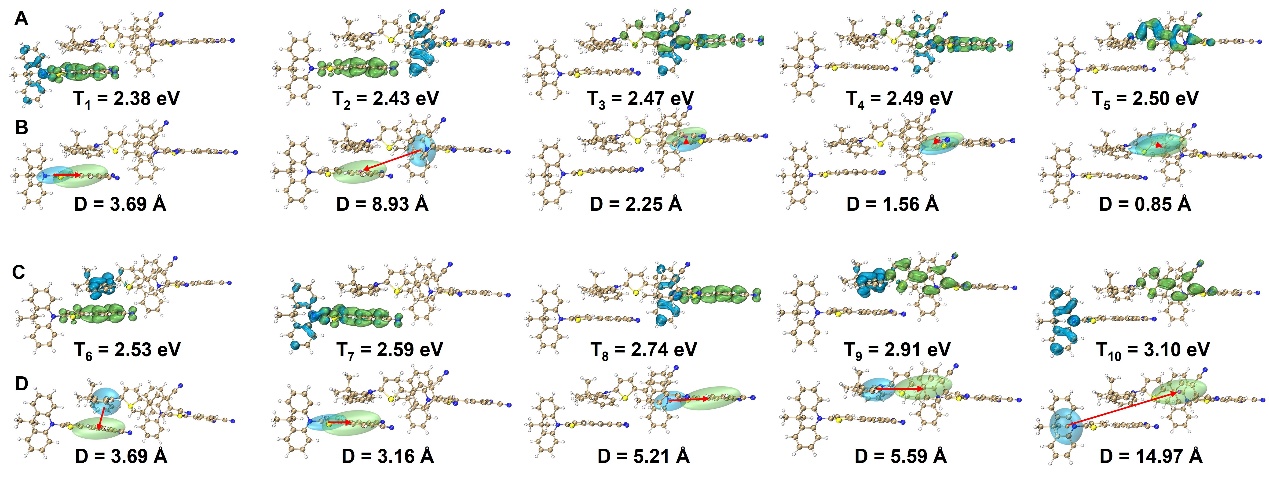


## **Figure S36.** TD-DFT calculation results of triplet excited state of trimeric M2

The isosurface maps of electron-hole distribution (A, C) and Cele-Chole distribution (B, D) of trimeric M2 at triplet excited state (T_1_-T_10_). Bule and green isosurfaces correspond to hole and electron distribution, respectively. Chole and Cele are the smooth distribution of holes (blue regions) and electrons (green regions). The orange ball and purple ball represent the hole and electron center of mass, respectively. The red arrows represent charge transfer from the hole to the electron center of mass. D is the distance between the hole and electron center of mass.

## **Figure S37.** Images of ROS-ID probe in 4T1 cancer cells under hypoxic and normoxic conditions

## **Figure S38.** Intracellular O_2_^‒•^ generation induced by M2

Fluorescence images of 4T1 cancer cells treated with DHE and M2 in normoxic (21% O_2_) or hypoxic (2% O_2_) atmosphere with or without laser irradiation (405 nm laser, power: 0.5%). [M2] = 20 μM; [DHE] = 10 μM. Red fluorescent channel: λ_ex_ = 510 nm, λ_em_ = 550-700 nm, scale bar: 20 μm.

## **Figure S39.** Workflow of the in vivo anti-tumor experiment

The 4T1 cancer cells were inoculated in mice to obtain 4T1 tumor-bearing mice, then M2 (5 mg/kg) was *in situ* injected into the tumor. After 4 hours, mice were irradiated for 20 minutes with white light illumination (30 mW/cm^2^). Finally, these mice were sacrificed for analysis.

## **Figure S40**. Photograph of dissected tumor tissues after 15-day treatment

# **Supplementary Tables**

## **Table S1.** Hyperparameters for the five supervised-learning models employed

| **Model** | **Parameter** |
| --- | --- |
| KNN | n_neighbors=6, weights='uniform', p=2, metric='euclidean' |
| SVM | C=100, kernel='rbf', gamma='scale' |
| NN | hidden_layer_sizes=(50,), activation='relu', solver='adam', alpha=0.0001, learning_rate_init=0.001 |
| RF | n_estimators=200, criterion='gini', max_depth=6, max_features='sqrt' |
| XGB | max_depth=6, learning_rate=1, n_estimators=100, objective='binary:logistic', booster='gbtree' |

## **Table S2.** Parameter settings of the UMAP algorithm used to project high dimension feature space into two dimensions for visualization

| **Parameter** | **Value** |
| --- | --- |
| n_components | 2 |
| min_dist | 0.5 |
| n_neighbors | 15 |
| metric | 'euclidean' |

## **Table S3.** Vertical excitation energies (eV) of S_1_-S_10_, T_1_-T_10_ excited states of M1 and M2

|  | M1 | M2 |
| --- | --- | --- |
| S_1_ | 2.05 | 2.32 |
| S_2_ | 2.84 | 3.11 |
| S_3_ | 3.42 | 3.72 |
| S_4_ | 3.66 | 3.76 |
| S_5_ | 3.80 | 3.84 |
| S_6_ | 3.83 | 3.94 |
| S_7_ | 4.13 | 3.99 |
| S_8_ | 4.18 | 4.19 |
| S_9_ | 4.19 | 4.35 |
| S_10_ | 4.21 | 4.42 |
| T_1_ | 2.04 | 2.30 |
| T_2_ | 2.48 | 2.47 |
| T_3_ | 2.83 | 3.10 |
| T_4_ | 2.97 | 3.33 |
| T_5_ | 3.35 | 3.34 |
| T_6_ | 3.42 | 3.67 |
| T_7_ | 3.58 | 3.70 |
| T_8_ | 3.65 | 3.72 |
| T_9_ | 3.66 | 3.73 |
| T_10_ | 3.69 | 3.78 |

## **Table S4.** HOMO-LUMO and Gibbs free energy calculation results of M1 and M2 at the B3LYP/6-31G(d) Opt level

|  | M1 | M2 |
| --- | --- | --- |
| HOMO (eV) | -5.16 | -5.39 |
| LUMO (eV) | -2.64 | -2.58 |
| Δ*E*_HOMO-LUMO_ (eV) | 2.52 | 2.81 |
| T_1_ (eV) | 2.04 | 2.30 |
| *E*_ox_ (vs. Ag/AgCl) (V) | 0.82 | 1.05 |
| *E*_ox_ (vs. NHE) (V) | 1.02 | 1.25 |
| ΔG_(PS-O2)_ (kcal/mol) | -18.91 | -19.83 |
| ΔG_(PS-HO_^-^_)_ (kcal/mol) | -35.58 | -27.11 |

**Calculation of Redox potential**

The oxidation potential (vs. Ag/AgCl) of M1 and M2 were calculated by the

*E*_ox_ = -4.80 + *E*_Fc/Fc+_ - *E*_HOMO/e_  Equation S2^[1-2]^

where *E_Fc/Fc_*_+_ is 0.46 V which is taken from the related reference. With Equation S2, the oxidation potential of M1 and M2 can be calculated based on the TD-DFT calculated HOMO energies.

**Calculation of Gibbs free energy**

The Gibbs free energy between M1/M2 and oxygen were calculated by the Rehm-Weller equation:

Δ*G* = *E*_ox_-*E*_re_-*E*_0-0_ Equation S3

*E*_ox_ and *E*_re_ are estimated from the oxidative potential (vs. NHE) of the donor (M1 and M2) and the reductive potential of the acceptor (oxygen). The *E*_ox_ of photosensitizers was calculated by using Equation S2. The *E*_re_ of ground state oxygen was -0.2 eV.^[3]^ *E*_0-0_ is the lowest excited state energy (proximate energy level of T_1_) of M1 and M2.

The Gibbs free energy changes for M1/M2 extracting an electron from microenvironmental hydroxyl radical were calculated by the TD-DFT calculation and the below equation^[4]^

^3^PS + OH^-^ → ^3^PS^‒•^ + HO• Equation S4

## **Table S5.** Crystal data and structure refinement for M2 single crystal^[5]^

| Formula | C_27_H_19_N_3_S | Density | 1.284 Mg/m^3^ |
| --- | --- | --- | --- |
| Formula weight | 417.51 | Z | 4 |
| Temperature (K) | 100.00 | Absorption coefficient | 0.169 mm^-1^ |
| Crystal system | Monoclinic | F(000) | 872 |
| Space group | *P*2_1_/n | Crystal size | 0.195 x 0.177 x 0.111 mm^3^ |
| a/Å | 10.2816(5) | Wavelength | 0.71073 Å |
| b/Å | 17.2645(8) | Reflections collected | 100044 |
| c/Å | 12.1747(5) | Data / restraints / parameters | 6620 / 0 / 282 |
| α/° | 90 | Independent reflections | 6620 [R(int) = 0.0972] |
| β/° | 91.256(2) | Goodness-of-fit on *F*2 | 1.062 |
| γ/° | 90 | Final *R* indices [I>2*σ*(*I*)] | *R*_1_ = 0.0466, *wR*_2_ = 0.1067 |
| Volume/Å^3^ | 2160.57(17) | *R* indices (all data) | *R*_1_ = 0.0607, *wR*_2_ = 0.1176 |

## **Table S6.** Vertical excitation energies (eV) of excited singlet state (S_1_-S_10_), and excited triplet state (T_1_-T_10_) of monomer, dimer and trimer of M2 optimized from M2 single crystal

|  | Monomer | Dimer | Trimer |
| --- | --- | --- | --- |
| S_1_ | 2.52 | 2.55 | 2.42 |
| S_2_ | 3.37 | 2.60 | 2.48 |
| S_3_ | 3.74 | 2.84 | 2.49 |
| S_4_ | 3.84 | 3.05 | 2.54 |
| S_5_ | 3.93 | 3.39 | 2.71 |
| S_6_ | 4.09 | 3.48 | 2.90 |
| S_7_ | 4.15 | 3.59 | 3.10 |
| S_8_ | 4.24 | 3.71 | 3.22 |
| S_9_ | 4.46 | 3.72 | 3.30 |
| S_10_ | 4.56 | 3.78 | 3.31 |
| T_1_ | 2.40 | 2.42 | 2.38 |
| T_2_ | 2.60 | 2.49 | 2.43 |
| T_3_ | 3.35 | 2.59 | 2.47 |
| T_4_ | 3.53 | 2.62 | 2.49 |
| T_5_ | 3.55 | 2.86 | 2.50 |
| T_6_ | 3.70 | 3.05 | 2.53 |
| T_7_ | 3.75 | 3.33 | 2.59 |
| T_8_ | 3.77 | 3.44 | 2.74 |
| T_9_ | 3.82 | 3.51 | 2.91 |
| T_10_ | 3.91 | 3.52 | 3.10 |

## **Table S7.** Energy gaps and spin-orbit coupling (SOC) constants of monomeric M2 at S_1_ → T_1_, T_2_ transitions

| Transition | Energy gaps (eV) | Spin-orbit coupling (cm^-1^) |
| --- | --- | --- |
| S_1_ → T_1_ | 0.12 eV | 1.10 |
| S_1_ → T_2_ | 0.08 eV | 0.99 |

## **Table S8.** Energy gaps and spin-orbit coupling (SOC) constants of dimeric M2 at S_1_ → T_1_, T_2_, T_3_, T_4_ transitions

| Transition | Energy gaps (eV) | Spin-orbit coupling (cm^-1^) |
| --- | --- | --- |
| S_1_ → T_1_ | 0.13 | 1.22 |
| S_1_ → T_2_ | 0.06 | 0.04 |
| S_1_ → T_3_ | 0.04 | 0.94 |
| S_1_ → T_4_ | 0.07 | 0.89 |

## **Table S9.** Energy gaps and spin-orbit coupling (SOC) constants of trimeric M2 at S_1_ → T_1_, T_2_, T_3_, T_4_, T_5_, T_6_, T_7_ transitions

| Transition | Energy gaps (eV) | Spin-orbit coupling (cm^-1^) |
| --- | --- | --- |
| S_1_ → T_1_ | 0.04 | 0.02 |
| S_1_ → T_2_ | 0.01 | 0.01 |
| S_1_ → T_3_ | 0.05 | 0.32 |
| S_1_ → T_4_ | 0.07 | 0.56 |
| S_1_ → T_5_ | 0.09 | 0.05 |
| S_1_ → T_6_ | 0.11 | 0.52 |
| S_1_ → T_7_ | 0.17 | 0.05 |

## **Table S10.** TD-DFT calculation results of excited states of monomeric M2

| **Transition** | **D (Å)** | **S_r_** | **t (Å)** | **Transition** | **D (Å)** | **S_r_** | **t (Å)** |
| --- | --- | --- | --- | --- | --- | --- | --- |
| S_0_ → S_1_ | 6.19 | 0.21 | 4.05 | S_0_ → T_1_ | 3.30 | 0.66 | 0.20 |
| S_0_ → S_2_ | 7.46 | 0.13 | 5.83 | S_0_ → T_2_ | 3.42 | 0.67 | 0.23 |
| S_0_ → S_3_ | 6.65 | 0.29 | 4.32 | S_0_ → T_3_ | 6.84 | 0.30 | 4.70 |
| S_0_ → S_4_ | 3.65 | 0.65 | 0.62 | S_0_ → T_4_ | 0.70 | 0.90 | -2.34 |
| S_0_ → S_5_ | 7.04 | 0.15 | 4.91 | S_0_ → T_5_ | 0.12 | 0.92 | -1.06 |
| S_0_ → S_6_ | 5.56 | 0.52 | 2.68 | S_0_ → T_6_ | 3.01 | 0.80 | 0.22 |
| S_0_ → S_7_ | 5.29 | 0.31 | 2.92 | S_0_ → T_7_ | 1.99 | 0.77 | -1.40 |
| S_0_ → S_8_ | 2.17 | 0.77 | -0.54 | S_0_ → T_8_ | 4.01 | 0.76 | 0.61 |
| S_0_ → S_9_ | 0.47 | 0.73 | -1.37 | S_0_ → T_9_ | 2.85 | 0.65 | 0.34 |
| S_0_ → S_10_ | 7.80 | 0.20 | 6.16 | S_0_ → T_10_ | 1.95 | 0.74 | -0.77 |

For TD-DFT calculation results, D index represents the distance between the hole and the electron center of mass. Sr index represents the overlap between electron and hole distributions, the larger the Sr index, the higher the degree of overlap between hole and electron. t index represents the degree of separation between the hole and the electron, positive t index implies that the hole and electron are separated sufficiently. For local excitation or global excitation, the excited states featured small D, large Sr, and obviously negative t. On the contrary, for charge transfer excitation, they often show large D, small Sr, and positive t.

## **Table S11.** TD-DFT calculation results of excited states of dimeric M2

| **Transition** | *D* (Å) | *S*_r_ | *t* (Å) | **Transition** | *D* (Å) | *S*_r_ | *t* (Å) |
| --- | --- | --- | --- | --- | --- | --- | --- |
| S_0_ → S_1_ | 6.09 | 0.21 | 3.85 | S_0_ → T_1_ | 2.92 | 0.69 | -0.03 |
| S_0_ → S_2_ | 3.71 | 0.12 | 2.34 | S_0_ → T_2_ | 0.98 | 0.84 | -1.66 |
| S_0_ → S_3_ | 6.05 | 0.22 | 3.94 | S_0_ → T_3_ | 3.67 | 0.17 | 2.27 |
| S_0_ → S_4_ | 14.96 | 0.01 | 12.87 | S_0_ → T_4_ | 3.73 | 0.62 | 0.63 |
| S_0_ → S_5_ | 3.77 | 0.16 | 2.62 | S_0_ → T_5_ | 5.51 | 0.38 | 2.94 |
| S_0_ → S_6_ | 7.42 | 0.14 | 5.74 | S_0_ → T_6_ | 14.95 | 0.01 | 12.88 |
| S_0_ → S_7_ | 6.70 | 0.07 | 4.16 | S_0_ → T_7_ | 3.22 | 0.42 | 1.82 |
| S_0_ → S_8_ | 2.42 | 0.45 | 0.59 | S_0_ → T_8_ | 5.21 | 0.52 | 2.42 |
| S_0_ → S_9_ | 7.35 | 0.18 | 5.51 | S_0_ → T_9_ | 1.63 | 0.84 | -1.73 |
| S_0_ → S_10_ | 5.14 | 0.23 | 1.96 | S_0_ → T_10_ | 0.87 | 0.88 | -2.78 |

## **Table S12.** TD-DFT calculation results of excited states of trimeric M2

| **Transition** | *D* (Å) | *S*_r_ | *t* (Å) | **Transition** | *D* (Å) | *S*_r_ | *t* (Å) |
| --- | --- | --- | --- | --- | --- | --- | --- |
| S_0_ → S_1_ | 9.00 | 0.03 | 6.81 | S_0_ → T_1_ | 3.69 | 0.62 | 0.67 |
| S_0_ → S_2_ | 3.84 | 0.11 | 2.00 | S_0_ → T_2_ | 8.93 | 0.03 | 6.69 |
| S_0_ → S_3_ | 6.23 | 0.20 | 4.00 | S_0_ → T_3_ | 2.25 | 0.42 | 0.53 |
| S_0_ → S_4_ | 3.74 | 0.11 | 2.35 | S_0_ → T_4_ | 1.56 | 0.57 | 0.00 |
| S_0_ → S_5_ | 6.16 | 0.21 | 4.03 | S_0_ → T_5_ | 0.85 | 0.84 | -1.88 |
| S_0_ → S_6_ | 6.03 | 0.23 | 3.87 | S_0_ → T_6_ | 3.69 | 0.16 | 2.26 |
| S_0_ → S_7_ | 14.97 | 0.01 | 12.87 | S_0_ → T_7_ | 3.16 | 0.69 | -0.05 |
| S_0_ → S_8_ | 8.98 | 0.02 | 7.27 | S_0_ → T_8_ | 5.21 | 0.45 | 2.39 |
| S_0_ → S_9_ | 3.78 | 0.16 | 2.26 | S_0_ → T_9_ | 5.59 | 0.36 | 2.97 |
| S_0_ → S_10_ | 3.73 | 0.16 | 2.32 | S_0_ → T_10_ | 14.97 | 0.01 | 12.88 |

# **Supplementary References**

[1] L. Cao, X. Lin, X. Liu, M. Wu, S. Liu, T. Wang, D. Mao, B. Liu, "Type-I Photosensitizer-Triggered Controllable Carbon Monoxide Release for Effective Treatment of Staph Skin Infection." *Nano Lett* **2023**.

[2] Z. Xie, Y. Xue, X. Zhang, J. Chen, Z. Lin, B. Liu, "Isostructural doping for organic persistent mechanoluminescence." *Nat Commun* **2024,** *15* (1), 3668.

[3] Y. Zhang, M. Zhao, J. Miao, W. Gu, J. Zhu, B. Cheng, Q. Li, Q. Miao, "Hemicyanine-Based Type I Photosensitizers for Antihypoxic Activatable Photodynamic Therapy." *ACS Mater. Lett.* **2023,** *5* (11), 3058-3067.

[4] M. Kang, Z. Zhang, W. Xu, H. Wen, W. Zhu, Q. Wu, H. Wu, J. Gong, Z. Wang, D. Wang, B. Z. Tang, "Good Steel Used in the Blade: Well-Tailored Type-I Photosensitizers with Aggregation-Induced Emission Characteristics for Precise Nuclear Targeting Photodynamic Therapy." *Adv Sci (Weinh)* **2021,** *8* (14), e2100524.

[5] Deposition numbers CCDC 2427761 contains the detailed crystallographic data of M2 in this paper. These data are provided free of charge by the joint Cambridge Crystallographic Data Centre and Fachinformationszentrum Karlsruhe [Access Structures](http://www.ccdc.cam.ac.uk/structures) service.
